# Assessing the use of automatic speech recognition and large language models for individuals with language impairments

**DOI:** 10.1038/s41467-026-76677-z

**Published:** 2026-09-10

**Authors:** Gelei Xu, Haoxinran Yu, Lixuan Wei, Yuxuan Liu, Dancheng Liu, Chenhui Xu, Jiajie Li, Ahmed Abbasi, Jinjun Xiong, Xiufan Yu, Zhi Zheng, Yiyu Shi, Ruiyang Qin

**Affiliations:** 1 University of Notre Dame, Notre Dame, IN USA; 2 State University of New York at Buffalo, Buffalo, NY USA; 3 Villanova University, Villanova, PA USA

**Keywords:** Health care, Computer science

## Abstract

Large language models (LLMs) have attracted much attention for healthcare applications, demonstrating strong potential in automating conversational interactions. However, cloud-hosted systems raise privacy concerns and text-based interfaces can be difficult for children, older adults, and individuals with language impairments. Edge-deployed, voice-enabled LLM systems may address these barriers by keeping data local and using automatic speech recognition (ASR) for speech input. However, ASR transcripts often contain disfluencies, fillers, grammatical errors, and recognition noise, which may reduce downstream LLM performance for speakers with atypical language patterns. Here, we systematically evaluate edge-oriented ASR-LLM pipelines for individuals with language impairments using comparison studies and ablation experiments across aphasia, child language impairment, and dementia datasets. We identify transcript errors, repetition, noise, and input length as factors affecting system performance and deployment feasibility. These findings highlight key challenges for building more reliable and accessible speech-enabled AI systems for healthcare applications.

## Abstract (web summary)

Speech-enabled AI systems may be less reliable for people with language impairments. Here, the authors evaluate ASR-LLM pipelines across aphasia, child language impairment and dementia datasets, identifying transcript errors, repetition, and length as key factors affecting performance.

## Introduction

The global healthcare sector is facing the crisis of staff shortage^1^. To address this challenge, innovators are leveraging cutting-edge Artificial Intelligence (AI) to bridge the gap between healthcare demand and accessibility^2,3^. Among these efforts, Large Language Models (LLMs) are being explored as a solution to address the bottleneck of conversational communication, a cornerstone of healthcare delivery. However, like most AIs, the performance of LLMs highly depends on the data used for model training. Given that very limited training data has been collected from people with language impairments, the efficacy of current LLMs for this population remains uncertain. This technical uncertainty poses the risk of significant disruptions and exclusion, particularly in vulnerable individuals, considering that more than 5% of the general population experiences communication impairments^4,5^.

Applying an LLM to healthcare communication for individuals with language impairments presents additional challenges beyond the model itself. First, many users, such as older adults living with dementia and children with language impairments, often face difficulties with text-based input, which remains the default interface for most LLMs. This limitation can hinder communication efficiency and reduce the effectiveness of care compared to traditional human-provided services^6^. Second, the interaction between users and LLMs often involves sensitive personal data, raising privacy concerns and potentially discouraging users from adopting LLM-driven healthcare solutions^7^. A promising approach to addressing the first challenge is the integration of Automatic Speech Recognition (ASR), which converts speech audio into text that can be processed by LLMs^8^. This ASR-LLM strategy has been adopted by major technology companies such as OpenAI^9^ and Google^10^. In our study, the term ASR-LLM refers to a pipeline where the LLM takes the transcript from the ASR as input, and produces a text response as output. To mitigate privacy concerns, deploying LLMs directly on edge devices, such as tablets or smartphones, offers a viable solution. By ensuring that all data processing occurs locally without cloud-based data exchange, edge-based LLMs can significantly reduce privacy risks and improve user trust^11,12^.

While deploying LLMs on edge devices and integrating ASR can improve accessibility and privacy, this combination introduces important practical challenges. Current ASRs convert speech into word-by-word text, often containing disfluencies, fillers (e.g., “um”, “hum”), and grammatical errors, issues that are particularly pronounced for individuals with language impairments. At the same time, on-device ASR models often undergo compression, which may degrade transcription quality. Similarly, LLMs deployed on edge devices are typically smaller and more resource-constrained than their cloud-based counterparts, resulting in reduced capacity to correct such transcription errors and perform robust reasoning^13,14^. Taken together, these factors raise an important question: how do ASR errors and reduced model capacity interact under edge deployment constraints, particularly for speakers with atypical language patterns? In particular, it remains unclear whether such constraints disproportionately affect individuals with language impairments, leading to differences in system performance compared to control populations. Addressing this question requires a systematic evaluation of ASR-LLM pipelines under realistic deployment conditions.

To investigate this question, we study ASR-LLM systems using clinical conversational datasets collected from three representative language impairment conditions: Aphasia, language impairments in children (LIC), and Dementia in older adults. Aphasia is a language disorder resulting from damage to one or more language-processing areas of the brain, often resulting from stroke. Approximately one-third of stroke cases lead to aphasia^15^. The selected LIC datasets include communication disorder that interferes with the development of language skills in children without hearing loss^16^, affecting about 1 in 14 children^17^. It also includes data recorded from children who suffer language impairments due to epilepsy. Dementia encompasses a group of conditions characterized by a progressive decline in language and cognitive functions, such as memory, reasoning, and problem solving. It is estimated that 4% of older adults aged 65 years and older have been diagnosed with dementia, with prevalence increasing with age^18^.

**Fig. 1:**
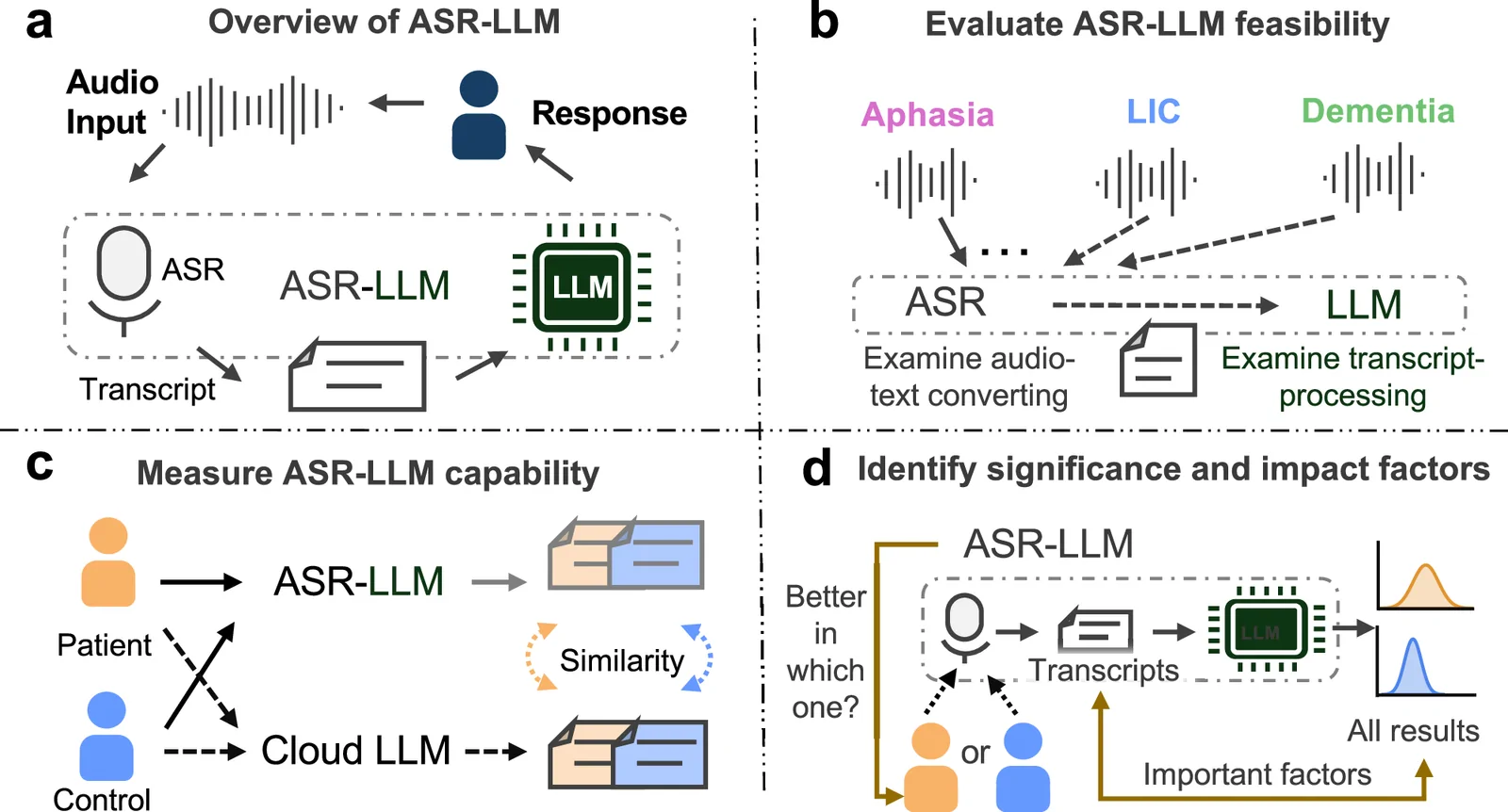
Overview of the study. **a** The ASR–LLM pipeline: the ASR model takes the audio input and produces a transcript, which is passed to the LLM to generate a response. **b** We evaluated the feasibility of the ASR–LLM pipeline across three clinical conditions (aphasia, LIC, and dementia) examining both the audio-to-text (ASR) and transcript-processing (LLM) stages. **c** We measured ASR–LLM capability for patient and control groups, using summaries generated by a cloud-based LLM from human transcripts as the reference and quantifying similarity to the ASR–LLM output. **d** We identified significant performance differences between patients and controls and the linguistic factors driving them.

We conduct large-scale experiments under controlled settings designed to approximate edge deployment constraints, complemented by targeted on-device validation on a physical edge platform (NVIDIA Jetson AGX). As illustrated in Fig. 1, the ASR converts audio into transcripts, which are then processed by an LLM to generate responses. To ensure robust evaluation, we adopt a multi-reference strategy using multiple state-of-the-art LLMs as references. The study consists of three main steps. First, we evaluate the deployment feasibility of ASR-LLM systems, focusing on whether ASR and LLM components can process the inputs under resource-constrained settings. Second, we measure the capability of edge ASR-LLM relative to a strong cloud-based LLM using text similarity metrics, allowing us to quantify both absolute performance and differences between patients and control groups. Finally, we assess whether these differences are statistically significant, and analyze the ASR-generated transcripts to identify linguistic factors that influence ASR-LLM performance.

In summary, the major contributions of this work are three-fold: (1) We present a large-scale empirical study of ASR-LLM systems for individuals with language impairments, covering multiple clinical populations, datasets, and a broad range of ASR-LLM model combinations under edge-oriented constraints. (2) We systematically characterize how linguistic factors (e.g., repetition, noise, and transcript length) affect ASR-LLM performance, and quantify performance disparities between patients and control groups across clinical conditions. (3) We provide a deployment-oriented analysis of ASR-LLM systems, highlighting the impact of resource constraints such as memory usage, latency, and input length, and identifying practical limitations for real-world edge deployment. We believe that this work provides both a systematic evaluation framework and practical insights for deploying ASR-LLM systems in healthcare settings, particularly for populations with language impairments.

## Results

We conduct a large-scale analysis to compare ASR-LLM performance between patients and control groups across multiple clinical conditions. Our study includes 20 TalkBank datasets spanning Aphasia (patient *N* = 227; control *N* = 210), language impairments in children (LIC; patient *N* = 341; control *N* = 444), and Dementia (patient *N* = 448; control *N* = 356). We evaluate 23 state-of-the-art LLMs combined with five Whisper ASR variants, resulting in 115 ASR-LLM configurations.

Performance is evaluated using three widely adopted semantic similarity metrics, ROUGE^19^, BERTScore^20^, and BLEURT^21^. As ground-truth summaries are not available in the datasets, we construct reference outputs using human-annotated transcripts paired with multiple strong cloud-based LLMs. Specifically, each ASR–LLM processes audio inputs to generate summaries, while reference summaries are generated from human transcripts using a set of high-capability LLMs under the same prompt setting. The prompt used throughout the study is

Please summarize the following transcript made by an experiment participant. Transcript:  < ASR-made/human annotator-made transcript > Summary:

Final evaluation scores are obtained by aggregating similarity metrics across these references, reducing dependence on any single model. We adopt summarization as the evaluation task because it requires holistic semantic understanding of the transcript and is sensitive to transcription errors, disfluencies, and linguistic irregularities in the input. Higher similarity between ASR-LLM outputs and the reference indicates better performance.

To compare patient and control groups, we apply non-parametric Wilcoxon tests with Bonferroni correction to account for multiple comparisons across model configurations.

### Characterizing deployment feasibility across hardware budgets

We begin with targeted experiments on physical edge hardware to examine how ASR–LLM systems behave under a high-memory on-device setting. These experiments were conducted on two NVIDIA Jetson AGX Orin 64GB platforms. Running the full 115-combination matrix on-device was computationally infeasible, so we designed a targeted evaluation strategy for each ASR–LLM combination. For each combination, we randomly selected three samples and additionally selected the three samples with the longest transcripts. This design allowed us to cover both routine on-device execution and more demanding long-context cases without requiring a full on-device evaluation of the entire model matrix.

**Fig. 2:**
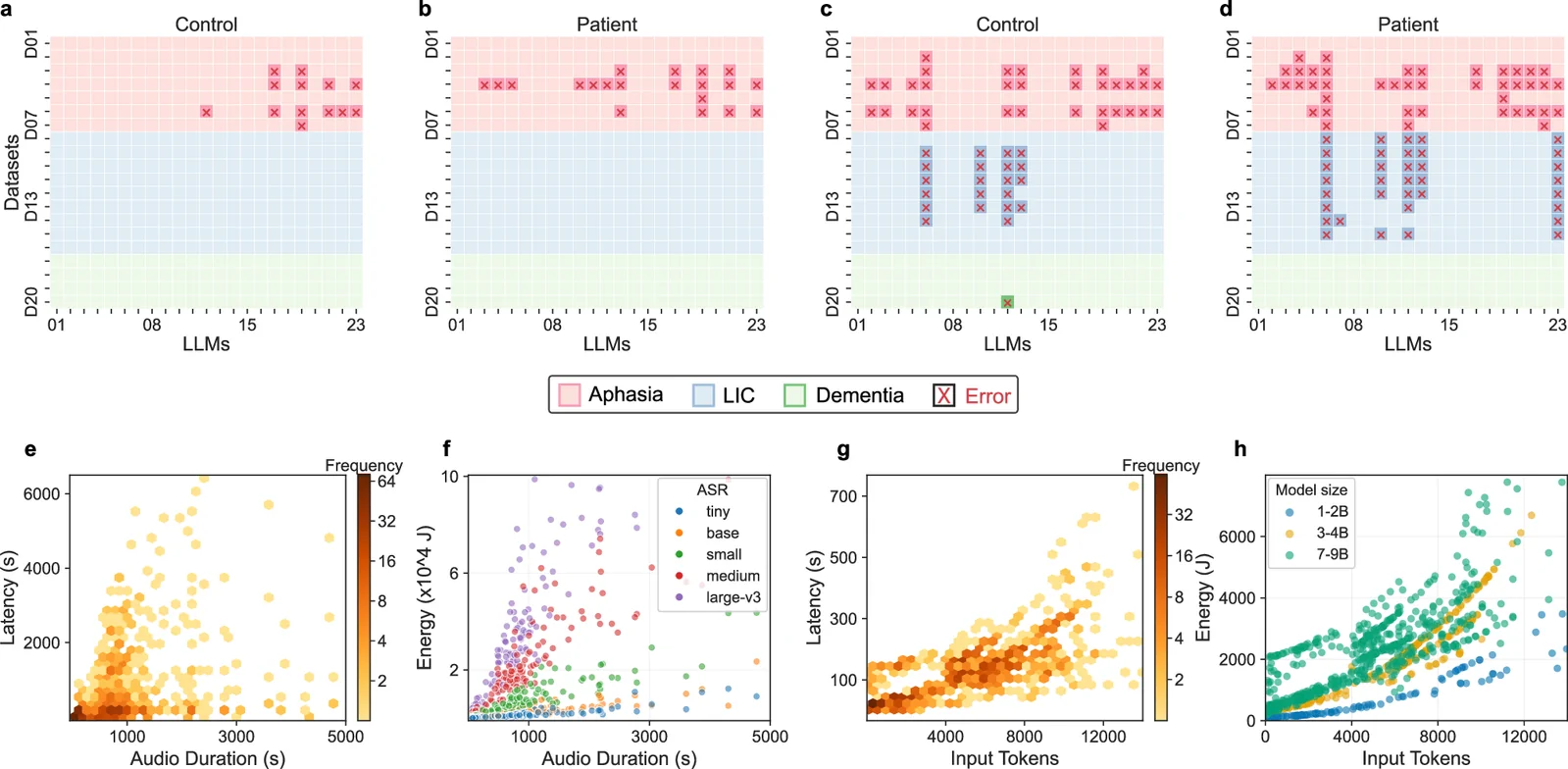
Deployment feasibility and cost of ASR–LLM pipelines under edge constraints. **a**, **b** On-device stress-test matrix on the NVIDIA Jetson AGX platform for control (**a**) and patient (**b**) groups; a grid cell is crossed if any selected long-context subset fails on that dataset. **c**, **d** Full-matrix screening in a 24 GB server setting across 20 datasets and 23 LLMs, each paired with five ASR variants, for control (**c**) and patient (**d**) groups; a cell is crossed if any ASR–LLM combination fails on that dataset. **e**, **f** Latency (**e**) and energy (**f**) measurements for the ASR stage on the edge, as a function of audio duration. **g**, **h** Latency (**g**) and energy (**h**) measurements for the downstream LLM stage on the edge, as a function of input length. Source data are provided as a Source Data file.

Across these on-device experiments, all tested ASR models successfully processed the audio inputs and produced transcripts. Failures arose at the downstream LLM stage. Figure 2a, b summarize these failure cases on the AGX platform across datasets. In the LIC and Dementia datasets, all tested combinations completed inference successfully on the AGX platform. In Aphasia, failures were concentrated in several datasets, including NEURAL, NEURAL_2, UNH, Richardson, and UMD, across multiple LLMs. The observed failure types were “CUDA out-of-memory” and “Probability tensor contains either inf, ‘nan’ or element  < 0”. We note that these failure patterns are also influenced by implementation choices and may be mitigated in part by memory-efficient techniques such as flash attention, gradient checkpointing, or context windowing. At the same time, Aphasia transcripts were typically longer than those in the other two conditions, which suggests that input length plays an important role in determining whether the downstream LLM can complete inference on-device. These observations motivate a closer analysis of runtime, energy consumption, and memory usage on the edge platform.

We next quantify the practical cost of on-device execution on the AGX platform for both stages of the ASR–LLM pipeline. As shown in Fig. 2e, f, the ASR stage becomes increasingly expensive as audio duration grows. ASR latency rises substantially for longer recordings, and the corresponding energy consumption also increases. In addition, ASR energy cost depends strongly on model size, with larger Whisper variants consuming substantially more energy than smaller ones for comparable audio durations. On the AGX platform, the energy required for audio transcription was often substantial. For Whisper-tiny and Whisper-base, the total energy consumption was generally below 10,000 J, whereas Whisper-medium and Whisper-large-v3 could easily exceed 20,000 J. These results show that transcription itself already imposes a significant deployment cost on edge hardware.

The downstream LLM stage shows a similar pattern with respect to transcript length, as shown in Fig. 2g, h. Latency increases markedly with input tokens, and long transcripts can require several minutes of inference for a single sample. Energy consumption also grows with input length and is strongly associated with LLM model size. Under comparable input lengths, larger models consistently consume more energy than smaller ones. For example, when the input length is around 4000 tokens, models in the 1B–2B range typically consume less than 500J, whereas models in the 7B–9B range often consume more than 2,000J. These results show that practical deployment cost accumulates across both stages of the pipeline. Audio duration drives the cost of ASR transcription, while transcript length drives the cost of downstream summarization. Even under a high-memory edge setting using the 64 GB NVIDIA Jetson AGX Orin platform, long recordings remain expensive in latency and energy. This motivates the next stage of analysis, in which we perform a full-matrix screening study under a lower and more standardized memory budget.

The on-device AGX experiments showed that long-context processing is strongly associated with practical deployment cost and failure risk, but running the full 115-combination matrix on physical edge hardware was computationally infeasible. We therefore performed a large-scale screening study in a lower-memory server setting, using a 24 GB GPU memory budget. Although this setting does not represent direct physical edge execution, it provides a controlled and scalable memory-constrained configuration for identifying deployment-relevant failure patterns across datasets and model combinations. It also better approximates a more constrained and more typical deployment budget than the 64 GB AGX platform, which represents a relatively high-end edge setting.

Under this 24 GB configuration, all tested ASR models successfully processed the audio inputs and produced transcripts. Failure cases arose at the downstream LLM stage, where some ASR-generated transcripts could not be processed successfully by certain LLMs. Figure 2c, d summarize these failure cases across datasets and ASR–LLM combinations. Failure cases were observed in both patient and control groups, although they occurred less frequently in controls. In Aphasia, failures were concentrated in several datasets, including NEURAL, NEURAL_2, and UNH, across multiple LLMs. In LIC, failures were concentrated in a smaller subset of LLMs across multiple datasets. These patterns suggest that downstream feasibility in a constrained memory setting depends on both model choice and transcript characteristics, and they motivate a more detailed analysis of how transcript length and model type shape failure behavior.

**Fig. 3:**
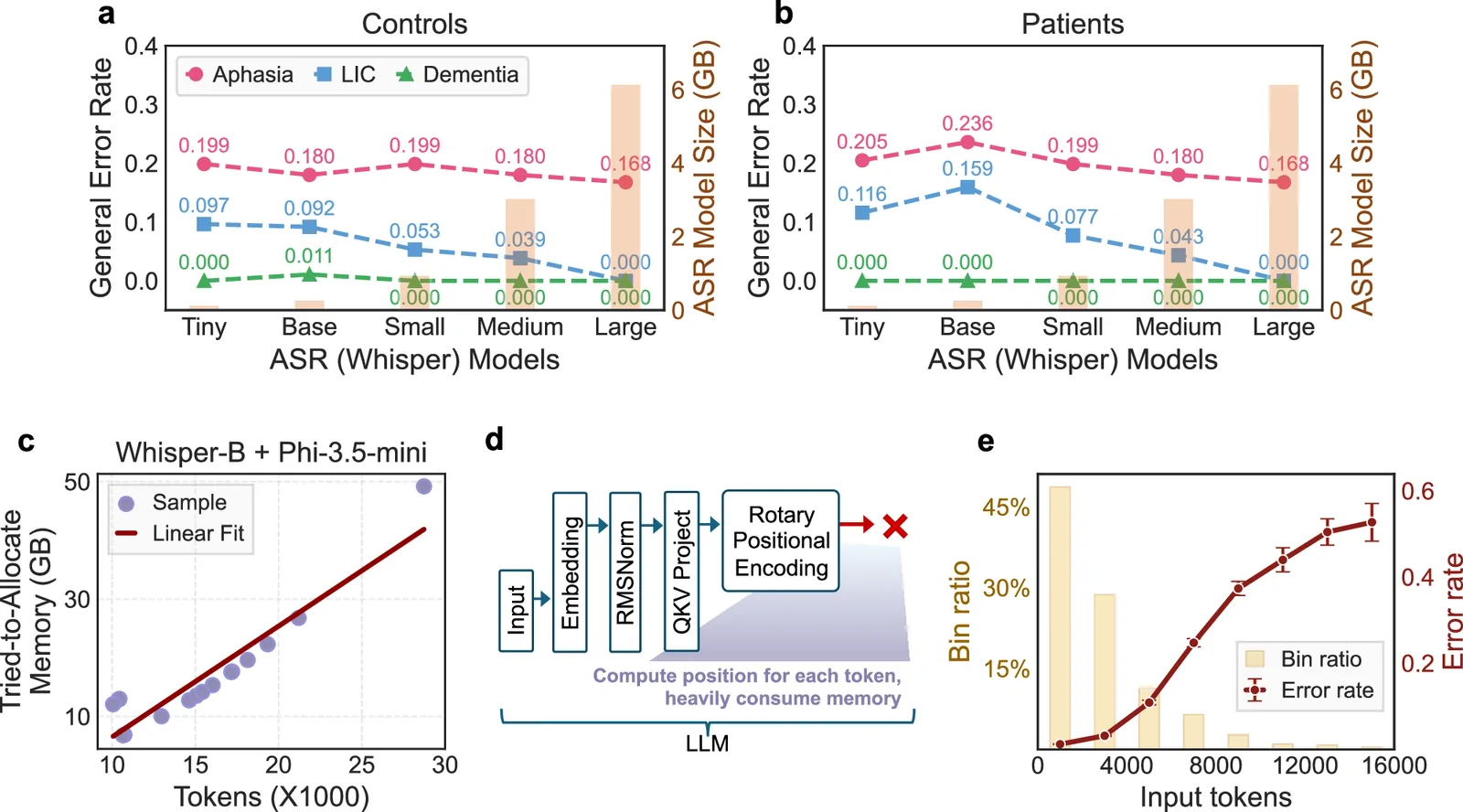
Factors associated with downstream failures in the lower-memory (24 GB) setting. **a**, **b** Relationship between downstream failure rate and ASR model size for controls (**a**) and patients (**b**); larger ASR models generally reduce failure rates. **c** A representative example showing that the memory required for inference increases with input length (Whisper-B + Phi-3.5-mini), with a linear fit. **d** Schematic of the LLM inference stage, indicating where memory-bound failure occurs during long-context processing. **e** Error rate versus input token length for edge LLM summarization. Each data point is one independent evaluation instance—one speech recording from one participant, processed by one ASR model and one edge LLM (*n* = 124,508 instances). Instances are grouped into 2,000-token bins; markers show the binomial error rate (mean of the binary error indicator) per bin, and error bars are Wilson score 95% confidence intervals. Per-bin sample sizes, from [0–2k) to [14k–16k), were *n* = 60,592; 35,751; 14,060; 8037; 3357; 1201; 1013; and 497. Bars show the proportion of instances per bin.

Because the failures arose after transcription, we next examined which properties of the upstream ASR output and downstream input were associated with this behavior. In particular, we analyzed how ASR model size and transcript length influenced downstream failure rates in the 24GB setting. Larger ASR models generally reduced downstream failure rates, as shown in Fig. 3a, b. Treating one ASR–LLM combination applied to one dataset as a trial, we measured the proportion of failed trials within each disease domain for each ASR size. In LIC, error ratios declined markedly with increasing ASR size for both patients and controls. In Aphasia, error ratios also declined with larger ASR models, with a stronger reduction in patients than in controls. These results suggest that upstream ASR quality influences downstream feasibility even though the failures themselves occur at the LLM stage.

Transcript length showed a strong association with downstream failure behavior. Figure 3c presents a representative example in which memory demand increases with input length. Figure 3d provides a schematic view of how long-context inference can place increasing pressure on the downstream LLM runtime. Figure 3e shows the population-level trend across token-length bins, where error rates increase monotonically for longer inputs. We note that these observed failure patterns are also influenced by implementation choices, and that memory-efficient techniques such as flash attention, gradient checkpointing, or context windowing may shift the practical boundary at which they arise. Even with this caveat, the broader trend is consistent. Longer transcripts place greater memory and numerical demands on downstream LLM inference and are therefore more likely to trigger failures under constrained deployment settings.

### Assess the ASR-LLM performances and the significant differences between patients and controls

Using three commonly used LLM performance metrics (ROUGE^19^, BERTScore^20^, and BLEURT^21^), the ASR-LLM median performances in Table 1 show clear differences across clinical conditions and between patient and control groups. Overall, the highest performance was observed in the Dementia datasets, whereas the lowest performance was generally observed in Aphasia, particularly for patient groups.

**Table 1:** The median performance of all feasible ASR-LLMs on patients and controls on metrics: ROUGE, BERTScore, Bleurt

		ROUGE		BERTSCORE		BLEURT	
	Dataset	Patients	Controls	Patients	Controls	Patients	Controls
**Aphasia**	Baycrest	0.35	0.39	0.85	0.86	−0.71	−0.64
	MSU	0.36	0.40	0.86	0.86	−0.69	−0.66
	NEURAL	0.34	0.42	0.86	0.86	−0.66	−0.66
	NEURAL_2	0.32	0.38	0.85	0.86	−0.79	−0.76
	UMD	0.32	0.38	0.85	0.86	−0.69	−0.65
	UNH	0.32	0.36	0.85	0.85	−0.80	−0.76
	Richardson	0.31	0.41	0.85	0.86	−0.80	−0.72
**LIC**	ENNI	0.37	0.39	0.86	0.86	−0.81	−0.78
	EllisWeismer/30ec	0.34	0.36	0.85	0.85	−0.79	−0.71
	EllisWeismer/30pc	0.32	0.34	0.85	0.85	−0.78	−0.75
	EllisWeismer/42ec	0.36	0.35	0.85	0.85	−0.72	−0.74
	EllisWeismer/42pc	0.35	0.35	0.85	0.85	−0.72	−0.71
	EllisWeismer/54ec	0.37	0.36	0.86	0.85	−0.66	−0.66
	EllisWeismer/54int	0.38	0.36	0.86	0.86	−0.68	−0.68
	EllisWeismer/66conv	0.37	0.37	0.86	0.86	−0.73	−0.72
	Poler	0.41	0.44	0.86	0.87	−0.66	−0.57
**Dementia**	ADReSS-IS2020	0.47	0.52	0.88	0.89	−0.45	−0.44
	Delaware	0.40	0.39	0.86	0.86	−0.72	−0.74
	Lu	0.45	0.53	0.88	0.90	−0.43	−0.30
	Pitt/cookie	0.50	0.54	0.89	0.89	−0.43	−0.40

The higher the values of the three metrics, the better the performances.

For ROUGE, control groups generally outperformed patient groups in Aphasia and Dementia. In contrast, the LIC datasets showed more mixed patterns, with some datasets favoring patients and others favoring controls. This suggests that performance disparities are more consistent in Aphasia and Dementia, while LIC exhibits greater heterogeneity across datasets. For BERTScore, the differences between patients and controls were smaller overall, typically on the order of 0.01 or less. Although controls still performed better in more cases, the margins were limited, indicating that semantic similarity remained relatively stable even when other metrics showed clearer group separation. For BLEURT, control groups generally achieved better scores than patient groups across all three clinical categories. This pattern was especially consistent in Aphasia, where every dataset showed higher BLEURT scores for controls. Compared with ROUGE and BERTScore, BLEURT appears more sensitive to the quality differences between patient and control summaries.

To assess the differences in performance between controls (C) and patients (P), we considered a two-sided hypothesis test focusing on the null hypothesis

1 $${H}_{0}:\, {{\rm{there}}}\; {{\rm{was}}}\; {{\rm{no}}}\; {{\rm{difference}}}\; {{\rm{in}}}\; {{\rm{ASR}}}{\mbox{-}}{{\rm{LLM}}}\; {{\rm{performance}}}\, \\ \,{{\rm{between}}}\; {{\rm{controls}}}\; {{\rm{and}}}\; {{\rm{patients}}}\,$$

and the two-sided alternative hypothesis

$${H}_{a1}:\,{{\rm{the}}}\; {{\rm{ASR}}}\mbox{-}{{\rm{LLM}}} \,{{\rm{performed}}}\; {{\rm{differently}}}\; {{\rm{between}}}\; {{\rm{controls}}}\; {{\rm{and}}}\; {{\rm{patients}}}.$$

For brevity, we denote the above two-sided test as

2 $${H}_{0}:P=C \quad \, {{\rm{versus}}}\, \quad {H}_{a1} :P \ne C$$

where “ = ” and “ ≠ ” represent equivalence and difference, respectively, in ASR-LLM performances.

The two-sided Wilcoxon test with Bonferroni-adjusted *p*-values (Table 2) showed systematic patient–control differences in several datasets, with the strongest and most consistent signals appearing in Aphasia. In this category, Baycrest, MSU, NEURAL, NEURAL_2, UNH, and Richardson all reached statistical significance in multiple metrics. LIC showed fewer globally consistent effects, but ENNI and Poler remained significant across ROUGE, BERTScore, and BLEURT, while EllisWeismer/30ec also showed significance across all three metrics. Dementia exhibited more dataset-specific effects, with Lu reaching significance and Pitt/cookie showing highly significant differences in ROUGE and BERTScore. The results provided significant evidence against the null hypothesis (*H*_0_), supporting the alternative hypothesis (*H*_1_).

**Table 2:** Bonferroni adjusted *p*-values from two-sided Wilcoxon tests on hypotheses (2) to assess the performance difference between patients and controls

	Dataset	*p*-value (ROUGE)	*p*-value (BERTSCORE)	*p*-value (BLEURT)
**Aphasia**	Baycrest	4.4e-02*	2.6e-02*	3.9e-02*
	MSU	9.5e-03*	2.9e-02*	3.3e-02*
	NEURAL	5.9e-08*	3.5e-09*	3.2e-05*
	NEURAL_2	6.1e-05*	2.7e-06*	1.4e-03*
	UMD	8.4e-02	6.6e-01	3.5e-01
	UNH	1.0e-01	9.7e-04*	3.1e-04*
	Richardson	2.4e-08*	5.5e-07*	8.0e-05*
**LIC**	ENNI	4.9e-05*	7.5e-06*	4.7e-04*
	EllisWeismer/30ec	6.5e-03*	7.8e-03*	9.9e-06*
	EllisWeismer/30pc	6.1e-02	3.2e-01	1.6e-02*
	EllisWeismer/42ec	9.3e-01	7.7e-01	5.5e-01
	EllisWeismer/42pc	5.7e-01	3.1e-01	2.1e-01
	EllisWeismer/54ec	5.8e-01	6.5e-02	1.0e+00
	EllisWeismer/54int	1.0e+00	1.0e+00	1.0e+00
	EllisWeismer/66conv	1.0e+00	1.0e+00	1.0e+00
	Poler	9.6e-08*	1.1e-07*	6.3e-07*
**Dementia**	ADReSS-IS2020	1.7e-01	5.1e-01	1.0e+00
	Delaware	1.0e+00	1.0e+00	1.0e+00
	Lu	9.4e-03*	3.1e-02*	2.2e-01
	Pitt/cookie	8.7e-12*	4.0e-07*	5.6e-02

*indicates statistical significance at the significance level 0.05 after Bonferroni correction. *p*-values exceeding 1 after Bonferroni correction were capped at 1.

### Assess the ASR-LLM preference: patients or controls

To further assess the directional differences in ASR-LLM performance, we considered two one-sided hypothesis tests to separately evaluate whether the performance is significantly better in patients than in controls, and vice versa. Specifically, one-sided Wilcoxon tests were employed to test the null hypothesis *H*_0_ (defined in (1)) against two one-sided alternative hypotheses

$${H}_{a2}: \,{{\rm{the}}}\; {{\rm{ASR}}}{\mbox{-}}{{\rm{LLM}}}\; {{\rm{performed}}}\; {{\rm{better}}}\; {{\rm{in}}}\; {{\rm{controls}}}\; {{\rm{than}}}\; {{\rm{in}}}\; {{\rm{patients}}}\,. \\ {H}_{a3}: \,{{\rm{the}}}\; {{\rm{ASR}}}{\mbox{-}}{{\rm{LLM}}}\; {{\rm{performed}}}\; {{\rm{better}}}\; {{\rm{in}}}\; {{\rm{patients}}}\; {{\rm{than}}}\; {{\rm{in}}}\; {{\rm{controls}}}\,.$$

Similarly, we denote

3 $${H}_{0}:P=C\quad \,{{\rm{versus}}}\,\quad {H}_{a2}:C > P$$

and

4 $${H}_{0}:P=C\quad \,{{\rm{versus}}}\,\quad {H}_{a3}:P > C$$

where “ > ” indicates superior ASR-LLM performance in one group compared to the other.

Bonferroni adjusted *p*-values from one-sided Wilcoxon tests on (3) and (4) were reported in Supplementary Table 1. Overall, statistically significant directional effects more often support *H*_*a*2_, indicating better performance in controls.

This pattern is most consistent in Aphasia datasets (Baycrest, MSU, NEURAL, NEURAL_2 and Richardson). UNH is a notable exception, as all three metrics support *P* > *C*, showing that patients outperform controls in this dataset. The LIC datasets show greater directional heterogeneity. ENNI, EllisWeismer/30ec, and EllisWeismer/30pc consistently support *C* > *P* across all three metrics, whereas Poler consistently supports *P* > *C*. Most of the remaining EllisWeismer subsets do not exhibit stable directional significance, indicating that group differences in LIC are more dataset-dependent. In Dementia, significant directional effects are more selective, but when present they favor controls. Lu supports *C* > *P* in ROUGE and BERTScore, and Pitt/cookie shows strong support for *C* > *P* across all three metrics. ADReSS-IS2020 and Delaware do not show significant directional differences.

### Assess the quality of ASR-generated transcripts: How they were related to ASR-LLM performance

The quality of ASR-generated transcripts impacted the ASR-LLM performance. To quantify transcript quality and assess its relation to ASR-LLM performance, four quality metrics were applied. Word Error Rate^22,23^ (WER) directly measured the proportion of transcription errors; higher scores indicated lower transcript accuracy, meaning the ASR made more mistakes during transcription. Repetition Ratio^24–26^ (RR) quantified vocabulary repetition; higher scores indicated greater levels of stuttering or disfluency presented in the audio. Type-Token Ratio^27,28^ (TTR) measured vocabulary diversity; higher scores indicated a richer vocabulary within the text. Since ASR may incorrectly recognize language fillers (e.g., hum, eh, etc.) as foreign-language words, TTR might also reflect ASR detection noise. Perplexity^29,30^ (PPL) reflected the semantic complexity of the text; higher scores indicate that the text was more difficult to understand.

**Fig. 4:**
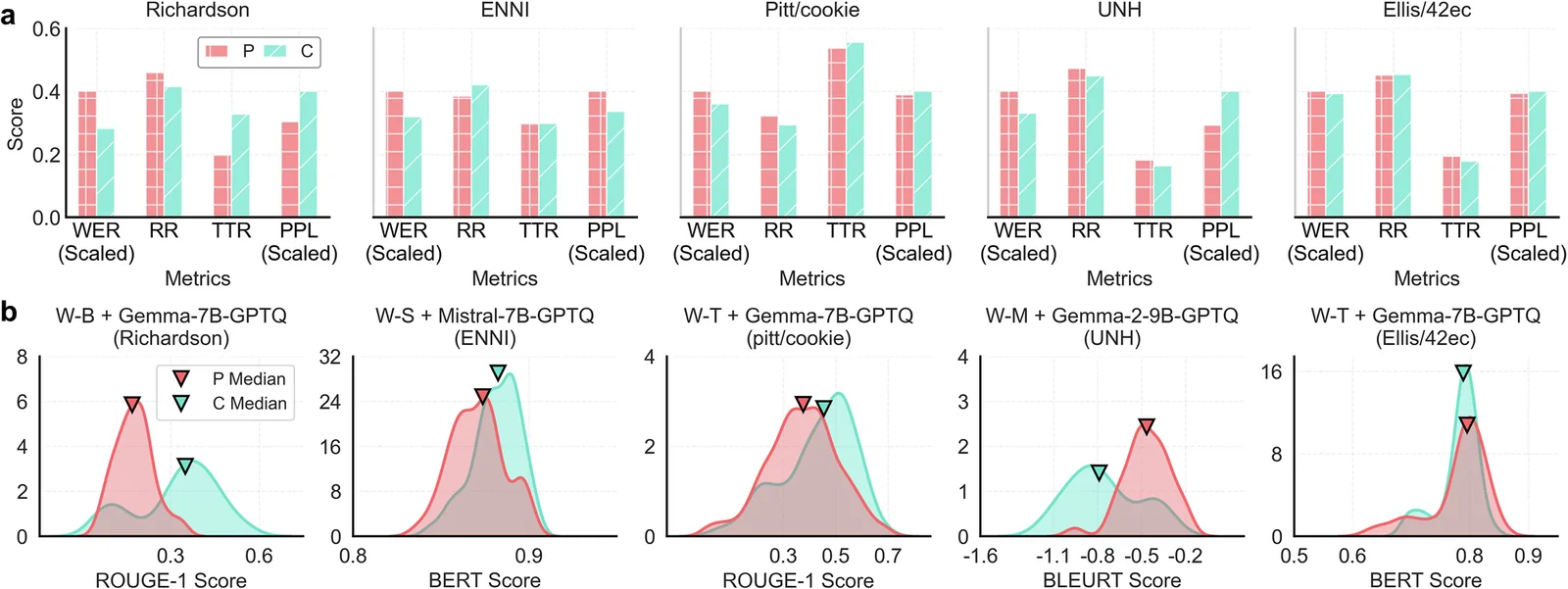
Transcript-quality metrics and ASR–LLM performance distributions across five representative datasets. **a** Average WER, RR, TTR, and PPL for ASR-generated transcripts on five datasets (Richardson, ENNI, Pitt/cookie, UNH, Ellis/42ec), shown for patient (P) and control (C) groups; WER and PPL were rescaled to the range 0 to 0.4 so they could share the y-axis with RR and TTR. **b** Performance distributions of exemplary ASR–LLM combinations on the same five datasets, with patient (P) and control (C) medians marked; the score shown (ROUGE-1, BERTScore, or BLEURT) is indicated on each x-axis. Source data are provided as a Source Data file.

Representative datasets: Richardson (Aphasia), ENNI (LIC), and Pitt/cookie (Dementia), UNH (Aphasia) and Ellis-42ec (LIC) were selected to demonstrate how the ASR-generated transcripts were reflected in the metrics. The WER, RR, TTR, and PPL scores computed for the ASR-generated transcripts from these five datasets are presented in Fig. 4a.

Across Richardson, ENNI, Pitt/cookie, and UNH, the average WER was higher in patients than in controls. This may suggest that ASR errors were not positively associated with ASR-LLM performance. In Ellis/42EC, there was no difference in WER between patients and controls, which was consistent with the patterns observed in RR, TTR, and PPL. Average RR scores were higher in Richardson (Aphasia), UNH (Aphasia), and Pitt/cookie (Dementia), due to the fact that increased vocabulary repetition was evident in these datasets. In Ellis/42ec (LIC), Average RR scores were roughly equivalent between patients and controls. This may explain the lack of significant ASR-LLM performance differences. Patterns of PPL appeared to align with those observed for RR. For TTR, average scores were higher in controls for Richardson (Aphasia) and Pitt/cookie (Dementia). This pattern was reversed in UNH (Aphasia). In Ellis/42ec (LIC) and ENNI (LIC), patients and controls exhibited similar TTR average scores.

### Verify the identified transcripts patterns to improve the ASR-LLM performance

To demonstrate representative transcript patterns from the five datasets analyzed in the previous section, one patient sample and one control sample with median ASR-LLM performance were selected, as shown in Fig. 4b. These selected samples are presented in Table 3 and Suppl. Table 2. In these sample transcripts, clear patterns of repetition and noise were observed.

**Table 3:** Examples in Richardson, ENNI, and Pitt/cookie dataset, illustrating how the ASR-LLM performance changed after removing identified issues in ASR-generated transcripts

	Richardson via Whisper-Base + Gemma-2B-GPTQ	
**Patient: 2053 tokens**		**ROUGE**
Original	Like, do you know you’re in a coma? [...] She’s three days late. [noise: MBC 뉴스 김지훈입니다]. Yep. [...] gets over the ball. [rep: It’s, It’s, [... It’s,…] It’s, It’s,] So what you’d be missing? you	0.1294
Remove Repetition	Like, do you know you’re in a coma? [...] She’s three days late. [noise: MBC 뉴스 김지훈입니다]. Yep. [...] gets over the ball. [...] So what you’d be missing? you	0.1764
Remove Noise	Like, do you know you’re in a coma? [...] She’s three days late. Yep. [...] gets over the ball. [rep: It’s, It’s, [... It’s,…] It’s, It’s,] So what you’d be missing? you	0.2470
Remove Both	Like, do you know you’re in a coma? [...] She’s three days late. Yep. [...] gets over the ball. So what you’d be missing? you	0.2709
**Control: 1120 tokens**		**ROUGE**
Original	Okay. So there was this boy. and he liked to play soccer. [...] on your second slice of bread. And then you put the two slices of bread together. and that’s it.	0.3118
	**ENNI via Whisper-Small + Mistral-7B-GPTQ**	
**Patient: 629 tokens**		**BERTScore**
Original	The elephant is playing with apple. [...] [noise: ac ydych chi’n gwybod i’r ffordd i’r ffordd i’r ffordd. Rwyf yn] [rep: ddweud y [... ddweud y…] ddweud y] He wants to go [...] [noise: 그래서, 이거 아삭한 것 같아요] [rep: www[... www…]www] They both got the envelope.	0.8685
Remove Repetition	The elephant is playing with apple. [...] [noise: ac ydych chi’n gwybod i’r ffordd i’r ffordd i’r ffordd. Rwyf yn] He wants [...] [noise: 그래서, 이거 아삭한 것 같아요] They both got the envelope.	0.8739
Remove Noise	The elephant is playing with apple. [...] [rep: ddweud y [... ddweud y…] ddweud y] He wants to go [...] [rep: www[... www…]www] They both got the envelope.	0.8820
Remove Both	The elephant is playing with apple. [...] He wants to go see the circus bunny again to get us a pony [...] They both got the envelope.	0.8917
**Control: 492 tokens**		**BERTScore**
Original	at swimming pool. They saw a ball in the water. It’s in a horse or a jolly. [...] The giraffe is trying to swim through the ball.	0.8950
	**Pitt/cookie via Whisper-Tiny + Gemma-7B-GPTQ**	
**Patient: 106 tokens**		**ROUGE**
Original	You see [...] [noise: Ja, jag vet inte.] Yeah, [...] and a little boys trying to get [rep: cookies jar up and [...] and the cookie jar up.] and there’s an official girl with him. [noise: And this is the bancher of what he had called it.] He looks like it’s going to fall. [rep: I’m going to... [...] I’m going to...] I’m going to borrow, [...] Okay, that’s fine.	0.4151
Remove Repetition	You see [...] [noise: Ja, jag vet inte.] Yeah, [...] and a little boys trying to get cookies up. and there’s an official girl with him. [noise: And this is the bancher of what he had called it.] He looks like it’s going to fall. I’m going to borrow, [...] Okay, that’s fine.	0.4528
Remove Noise	You see [...] Yeah, [...] and a little boys trying to get cookies up. and there’s an official girl with him. He looks like it’s going to fall. [rep: I’m going to... [...] I’m going to...] I’m going to borrow, [...] Okay, that’s fine.	0.4716
Remove Both	You see [...] Yeah, [...] and a little boys trying to get cookies up. and there’s an official girl with him. He looks like it’s going to fall. I’m going to borrow, [...] Okay, that’s fine.	0.5094
**Control: 83 tokens**		**ROUGE**
Original	There’s a girl waiting to get a cookie from I guess her brother chairs tip and over where he’s in. He’s in the cookie jar and they cover it. [...] Windows open.	0.4999

Segments labelled [noise:…] were identified as recognition noise and [rep:…] as repetition; [...] indicates omitted transcript content. Values in the rightmost column are ROUGE or BERTScore, as indicated in each block header.

In Table 3, removing repetitions and noise from patient transcripts improved ASR-LLM performance. Notably, removing noise led to greater performance improvements than removing repetitions alone, suggesting that noise had a stronger negative impact on the LLM. After removing the language issues, ASR-LLM performances were much closer between patients and controls.

In the example of UNH dataset in Supplementary Table 2, repetitions and noise were present in both patient and control transcripts. While removing these factors from the control transcript improved ASR-LLM performance, the resulting performance (BLEURT: −0.3854) remained significantly lower than that on the patient sample (BLEURT: −0.2599). The control transcript contained substantially more tokens than the patient transcript (5109 vs. 1209). As shown in Supplementary Table 2, truncating the control transcript to match the length of the patient transcript (1209 tokens) further improved performance.

In the example of Ellis/42ec dataset in Supplementary Table 2, the lengths of the control and patient transcripts were comparable. After removing language pattern issues, both the patient’s example and the control’s example improved, and were still comparable. These findings were consistent with our previous analyses, suggesting that (1) repetition and noise degrade LLM performance and (2) comparable transcript lengths between patient and control groups contribute to similar performance levels.

## Discussion

This study provides a large-scale empirical evaluation of ASR–LLM systems across three clinically distinct populations with language impairments. The observed performance differences between patients and controls were clearly condition-dependent. Aphasia showed the most consistent group gaps, LIC showed the greatest heterogeneity across datasets, and Dementia achieved the highest overall scores while exhibiting more selective patient–control differences. These patterns suggest that ASR–LLM robustness does not transfer uniformly across clinical populations. They also indicate that the interaction between ASR output and downstream LLM summarization is shaped by the linguistic characteristics of each condition. Repetition, noise, and transcript length were all associated with lower downstream performance, with transcript length emerging as a particularly important factor. Larger ASR models generally reduced downstream failure rates, which suggests that improved transcription quality can help. At the same time, the persistence of group differences even with stronger ASR models indicates that downstream LLM limitations remain an important part of the problem.

The results also clarify the practical meaning of edge deployment in this setting. The AGX experiments provided direct on-device evidence under a high-memory edge platform, while the 24GB server experiments provided a scalable lower-memory screening setting. Across both settings, long-context processing emerged as a common bottleneck. In the AGX experiments, long recordings remained costly in latency and energy even when many combinations completed successfully. In the lower-memory full-matrix screening, the same pressure appeared more frequently as observable downstream failures. These findings suggest that deployment feasibility is governed by a resource-dependent boundary shaped by transcript length, model choice, runtime cost, and available memory.

Several limitations should be noted. Although we replaced the original single-model reference with a multi-LLM reference and observed high agreement across reference models, the evaluation still depends on generated summaries instead of human-authored gold summaries. In addition, the present study evaluates off-the-shelf systems under edge-oriented deployment constraints and does not include task-specific adaptation or personalization. A further limitation concerns the interpretation of statistical significance. Several patient–control differences were statistically significant across datasets and model settings, but statistical significance should not be treated as equivalent to clinical meaningfulness. The practical importance of these differences depends on the downstream clinical context, the magnitude of the effect, and whether the resulting transcripts or summaries would influence documentation, interpretation, or care workflows. Establishing clinical meaningfulness will require additional validation through clinician-centered assessment, task-specific clinical criteria, or prospective workflow studies.

### Ethical considerations

The performance gaps observed between patients and controls raise an important ethical concern for healthcare AI deployment. Systems intended to support communication in clinical settings may perform less reliably for the populations that are most likely to benefit from them^31,32^. In our study, ASR–LLM performance differed between patient and control groups across multiple datasets, which indicates that these systems are not equally robust across populations. This pattern is consistent with prior work showing that speech and language technologies trained predominantly on typical speech often generalize poorly to atypical or impaired speech, in part because such populations remain underrepresented in training data^33^.

These disparities have direct clinical implications. Lower-quality ASR transcripts and summaries may distort symptom descriptions, reduce the reliability of clinical documentation, and obscure changes that are meaningful for assessment or treatment. This issue is especially important in language impairment settings, where disfluency, repetition, and reduced lexical diversity are themselves clinically relevant signals. If such signals are handled poorly by the ASR–LLM pipeline, the resulting output may misrepresent the patient’s communication and introduce bias into downstream clinical workflows.

These findings suggest that the responsible deployment of ASR–LLM systems in healthcare requires population-specific evaluation before clinical use. Performance should be audited separately for patient and control groups, and deployment decisions should account for condition-specific differences in robustness^34^. Human oversight should remain an essential part of any clinical workflow that uses AI-generated transcripts or summaries, particularly for individuals with communication impairments^35^. More broadly, the field would benefit from benchmark datasets that better represent diverse language impairment profiles, severity levels, and demographic backgrounds^33^. The TalkBank resources used here provide an important starting point, but broader dataset development will be necessary for equitable and clinically reliable deployment.

### Personalization and future directions

The performance gaps observed in this study suggest that user-specific adaptation may be an important direction for improving ASR–LLM systems, especially for individuals with language impairments. Speech patterns in these populations are highly heterogeneous, and a pipeline tailored to a user’s linguistic profile may improve both transcription quality and downstream summarization. In this sense, personalization may be particularly beneficial for the groups that are currently the most difficult to serve reliably.

At the same time, personalization remains challenging under the present edge deployment setting. Our edge experiments show that long-context inference already places substantial demands on memory, latency, and energy. This makes additional adaptation steps difficult to support on current hardware. A second challenge is data availability. In realistic edge settings, a device may observe only a limited amount of user-specific speech, which makes continual learning and stable adaptation difficult in practice.

For these reasons, the most suitable form of personalization remains an open question. PEFT-based methods such as LoRA or adapters are promising directions^36,37^, particularly when larger curated datasets or privacy-preserving training strategies are available^38,39^. Few-shot and in-context adaptation are also appealing because they may allow limited personalization without modifying model parameters^40^. Future work should examine which adaptation strategies can most effectively improve robustness for language-impaired users while remaining compatible with the practical constraints of edge deployment. Broader benchmark resources that better represent diverse language impairment profiles will also be important for evaluating these approaches under clinically relevant conditions^33^.

## Methods

### Preparing datasets for the ASR-LLM study

We included 2026 participants from the Talkbank database^41^. As shown in Suppl. Table 3, Aphasia had seven datasets, including 227 patients and 210 controls in total; LIC had nine datasets, including 341 patients and 444 controls; Dementia had four datasets, including 448 patients and 356 controls. The dataset was selected only if it contained the patient and control samples. And each sample had the corresponding transcript made by professional human annotators. All datasets included the participant and the investigator. The participant could be either the patient or the control. The investigator was a doctor or a person related to clinical investigation.

The Aphasia datasets involved participants describing a story or a picture, with the investigator providing an initial prompt and limited further involvement. The Dementia datasets used similar methods, with the investigator guiding the conversation at the beginning and allowing participants to speak freely. The LIC datasets recorded active interactions between the investigator and the child participant throughout the session. These included play, toy descriptions, and storytelling tasks designed to induce natural language from the child.

In our study, each audio in each dataset was converted into a transcript via the ASR models. During the conversion, while the audio could include the participant and the investigator, the participant’s part was not split from the investigator’s to avoid context loss. Additionally, since the original recordings were professionally designed for clinical studies within TalkBank and featured utterance-aligned transcripts directly linked to audio media, splitting the audio could compromise the integrity of the data^41^.

### Workstation settings

This study used two complementary hardware settings. Targeted on-device validation was first conducted on two NVIDIA Jetson AGX Orin developer kits, each equipped with 64 GB of unified memory. These edge experiments were designed to assess practical deployment behavior under a high-memory edge configuration, including inference feasibility, latency, energy consumption, and memory usage.

To complement the on-device analysis, the full-matrix evaluation across 115 ASR–LLM combinations was conducted on the GPU cluster, with each job allocated a single NVIDIA A10 GPU with 24 GB VRAM. This configuration provided a standardized and more constrained memory budget for large-scale screening across datasets and model combinations.

All experiments were implemented in Python using PyTorch and the Hugging Face ecosystem.

### Building up the proper ASR-LLM

As illustrated in Suppl. Fig. 1, the ASR–LLM evaluation framework consists of two parallel paths. In the main pipeline, audio is first transcribed by the ASR model and the generated transcript is then summarized by the downstream edge LLM. In the reference pipeline, the human-annotated transcript is summarized by multiple cloud-based reference LLMs using the same prompt template. ROUGE, BERTScore, and BLEURT are computed between the ASR–LLM summary and each reference summary, and the resulting scores are aggregated to obtain the final evaluation score.

To enable audio input for the LLM, an ASR model was used to convert the audio into a transcript. In the ASR-LLM pipeline, the transcript was automatically forwarded to the LLM, which then generated a response based on the transcript. Three aspects required explicit definition: (1) ASR model selection, (2) LLM selection, and (3) default settings.

For ASR model selection, we compared several widely used ASR models, including Canary-1B^42^ (NVIDIA), Whisper-tiny, Whisper-medium, Whisper-v3 (OpenAI)^43^, Parakeet-RNNT-1.1B^44^ (NVIDIA), Wav2Vec2-base and Wav2Vec2-conformer^45^ (Meta), and SeamlessM4T^46^ (Meta). These ASR models were each paired with the same downstream LLM, Phi-3.5-mini-instruct, to generate text from audio on three representative datasets, Baycrest for Aphasia, Poler for LIC, and Delaware for Dementia. As shown in Suppl. Fig. 2, the Whisper family showed the most stable and competitive performance overall across the three datasets. SeamlessM4T performed competitively in some cases, but did not consistently outperform the Whisper models across datasets. Wav2Vec2-conformer also showed higher variability across samples, especially in patient groups. Therefore, Whisper models were selected for use in this study.

For LLM selection, five LLM families were included: Gemma (Google), Mistral (Mistral AI, France), Phi (Microsoft), Llama (Meta), and Qwen (Alibaba, China). These LLMs are widely used, with each having over 100,000 downloads on Hugging Face. Their model sizes were below 20 GB, allowing them to run on commonly available hardware such as NVIDIA Jetson Orin or NVIDIA Jetson AGX. For each LLM family, compressed versions based on quantization were also included.

There were two types of LLMs being tested: original LLMs and compressed LLMs, as shown in the Suppl. Table 4. For original LLMs, they were trained from scratch. Their parameter size was initialized to be small. For compressed LLMs, they were compressed from the original LLMs. The parameter size of compressed LLMs could be initialized to be large. They were compressed by lowering the precision of parameters, so to lower to resource usage. The compression technique used GPTQ^47^, a widely used LLM compression method. For all tested models, GPTQ could be well compatible with them. When we used the compressed LLMs, their parameter precision was set to 4-bit integer, with the activations as 16-bit floating-point (FP16).

For the default settings used to construct the ASR–LLM pipeline, we applied a unified configuration across all experiments to ensure fair comparison across model combinations, and compatibility with edge-oriented deployment constraints. The ASR sampling rate was fixed at 16,000 Hz, following Whisper’s pretraining configuration^43^. For the LLM, no input length limit was imposed in order to preserve the full conversational context of each transcript, since truncation could remove clinically meaningful information and introduce bias in patient–control comparisons. The LLM output was capped at 100 tokens to produce concise summaries while keeping generation cost bounded.

For decoding, we used *do*_*sample* = True, *temperature* = 0.1, *top*_*p*_ = 0.9, and *top*_*k*_ = 10. This configuration was chosen to encourage stable and near-deterministic generation while avoiding repetition artifacts that can arise under purely greedy decoding, especially for long transcripts^48^. For compressed models, we used GPTQ 4-bit quantization with FP16 activations as a practical memory–quality trade-off for edge-oriented inference. Batch size was set to 16 in the server-based large-scale experiments and reduced to 1 for on-device evaluation on the Jetson AGX platform due to memory constraints.

To verify that the decoding configuration did not unduly affect the main conclusions, we further conducted a sensitivity analysis over multiple *temperature*/*top*_*p*_ settings, as reported in Suppl. Table 5. Across representative models and datasets from all three disease categories, BERTScore remained highly stable, with maximum range ≤ 0.01, while ROUGE and BLEURT showed only modest variation under more extreme settings. These results indicate that the selected configuration provides a conservative and robust setting for stable generation.

### Measuring the ASR-LLM performance

The Aphasia, LIC, and Dementia datasets only contained the transcript made by the human annotator and the audio for each sample. To evaluate ASR–LLM performance, we compare summaries generated from ASR transcripts with reference summaries generated from the human transcripts. In the ASR–LLM pipeline, the audio is first converted into an ASR-generated transcript and then summarized by the downstream LLM. For reference generation, the human-annotated transcript is provided directly to a set of high-capability cloud-based LLMs. In both cases, the same prompt template is used

Please summarize the following transcript made by an experiment participant. Transcript:  < ASR-made/human annotator-made transcript > Summary:

We adopt summarization as the evaluation task because it requires holistic semantic understanding of the transcript and is sensitive to transcription errors, disfluencies, and linguistic irregularities in the input. Producing a summary requires the LLM to integrate information across the full conversation, which makes this task more suitable for assessing downstream understanding than tasks based on isolated spans or short responses. Summarization is also clinically relevant for our setting, since patient–clinician conversations are commonly distilled into concise notes, and it enables a uniform evaluation protocol across heterogeneous TalkBank datasets without requiring additional task-specific annotations^49–52^.

To reduce dependence on any single reference model, we construct a multi-LLM reference using four strong cloud-based LLMs, Claude, GPT-4o, GPT-5.2, and Llama. For each sample, each reference model generates a summary from the human transcript using the same prompt setting. We then compute ROUGE, BERTScore, and BLEURT between the ASR–LLM summary and each reference summary separately, and average the resulting scores to obtain the final evaluation score. Higher similarity indicates better ASR–LLM performance, as it suggests that the ASR–LLM output remains closer to reference summaries derived from the human transcript.

We use three complementary metrics to evaluate summary similarity. (1) ROUGE^19^ measure the token-to-token similarity. It computes the overlap between the LLM-produced output and the reference based on n-grams, sequences, and word pairs, providing a straightforward and interpretable estimate of the textual similarity. (2) BERTScore^20^ extends beyond token-level matching by leveraging contextual embeddings from BERT^53^. It calculates the cosine similarity between the contextual representations of the output and the reference, thus reflecting deeper semantic alignment. (3) BLEURT^21^ uses pretrained and fine-tuned BERT models to produce a more human-like judgment of text quality. By adapting BERT to the domain-specific data, BLEURT often better aligns with human evaluations compared to traditional methods. Collectively, these three metrics provide complementary views of summary quality, covering lexical overlap, semantic similarity, and learned human-aligned evaluation. This choice is also consistent with recent evaluation trends in impaired-speech and accessibility benchmarks, which increasingly emphasize semantic quality beyond word-level matching^8^.

To further examine the robustness of the multi-reference protocol, we computed pairwise correlations between evaluation scores derived from different reference models. As shown in Suppl. Fig. 3, agreement across reference models is consistently high, with Pearson correlations ranging from approximately 0.89 to 0.98 across ROUGE and 0.95 to 0.99 across BERTScore for both patient and control groups. This indicates that evaluation outcomes are not overly dependent on any single reference model and supports the use of the aggregated multi-LLM reference in the main analysis.

### Measuring the quality of the ASR-generated transcripts

ASR-generated transcripts serve as the direct input to the downstream LLM and can therefore contribute to performance differences in the ASR–LLM pipeline. State-of-the-art ASRs directly convert a speech audio word-by-word into text, which often includes noise, grammar issues (e.g., repeated words), and speech fillers (e.g., “uh”, “hmm”). These properties are especially relevant in language impairment settings, where transcript quality may affect how effectively the downstream LLM can extract and summarize the underlying semantic content.

To characterize transcript quality, we use four complementary metrics that capture different aspects of the ASR output as an intermediate representation. Word Error Rate (WER) measures transcription accuracy against the human-annotated transcript. Perplexity (PPL) measures how well a language model predicts a given text, with lower values indicating more fluent and predictable language^54^. Type-Token Ratio (TTR), defined as the ratio of unique tokens to total tokens^28^, measures lexical diversity and can reflect constrained language use or word-finding difficulty. Repetition Ratio (RR) quantifies the proportion of repeated tokens among unique token types in the transcript^25^, capturing perseveration and other repetition-related irregularities.

We selected these metrics because they assess transcript quality beyond recognition accuracy alone. WER captures whether the ASR system recognizes the spoken words correctly, whereas PPL, TTR, and RR characterize linguistic properties of the transcript that may influence downstream LLM behavior. This is particularly important in clinical speech, where atypical fluency, reduced lexical diversity, and repetition may persist even when word-level recognition appears acceptable. This choice is also consistent with recent work arguing that WER alone is insufficient for evaluating ASR in clinical and atypical speech settings^38^. In addition, these metrics can be computed consistently across all TalkBank datasets without requiring additional task-specific annotations. Example computations for these metrics are provided in Table 4. We also note that alternative metrics such as CER, phonetic similarity, and semantic robustness measures have been proposed in the literature^55–57^, but were not adopted here because our focus is on transcript properties that can be computed consistently across all TalkBank datasets and related directly to downstream LLM performance.

**Table 4:** Comparison of Linguistic Metrics for ASR Transcripts

Metric	Example Transcript	Computation and Values
**PPL**	**Fluent:** I went to the store and bought apples. Then I met my friend at the cafe. **Impaired:** Uh... I, uh, went... uh, to, um, the store... and, uh, uh, got some, um, uh, apples.	PPL = *e*^Loss^, computed using a language model where loss is the negative log-likelihood of the input text. **Fluent:** 18.2 (lower = more structured language) **Impaired:** 78.5 (higher = less predictable, fragmented speech)
**TTR**	**Fluent:** I went to the store and bought apples. Then I met my friend at the cafe. **Impaired:** Uh... I, uh, went... uh, to, um, the store... and, uh, uh, got some, um, uh, apples.	${{\rm{TTR}}}=\frac {{{\mbox{Unique}}}\, {{\rm{Tokens}}}}{{{\rm{Total}}}\,{{\rm{Tokens}}}}$ **Fluent:** 15/18 = 0.83 (high = diverse vocabulary) **Impaired:** 14/32 = 0.44 (low = frequent word repetition)
**RR**	**Fluent:** I went to the store and bought apples. Then I met my friend at the cafe. **Impaired:** Uh... I, uh, went... uh, to, um, the store... and, uh, uh, got some, um, uh, apples.	${{\rm{RR}}}=\frac{{{\rm{Repeated}}}\, {{\rm{Token}}}\,{{\rm{Types}}}\, }{{{\rm{Unique}}}\, {{\rm{Token}}}\, {{\rm{Types}}}}$ **Fluent:** Repeated token types : 3/15 = 0.20 (low = less repetition) **Impaired:** Repeated token types : 4/14 = 0.29 (high = more repetition)
**WER**	**Reference:** I went to the store and bought apples. **ASR Output (Fluent):** I went to the store and bought apples. **ASR Output (Impaired):** I go store bought apple.	${{\rm{WER}}}=\frac {S+D+I}{N}$ where *S* = substitutions, *D* = deletions, *I* = insertions, and *N* = reference word count. **Fluent:** 0/8 = 0.00 (perfect ASR match) **Impaired:** (2 subs + 3 deletion)/8 = 0.63 (high = more ASR errors)

For each metric, an example of fluent and impaired speech is shown alongside its computation and resulting value.

### Reporting summary

Further information on research design is available in the Nature Portfolio Reporting Summary linked to this article.

## Supplementary information

Supplementary Information

Peer Review file

Reporting Summary

## Source data

Source Data

## Data Availability

All datasets used in this study are available in the TalkBank database. The AphasiaBank datasets are available at https://talkbank.org/aphasia/access/English/Protocol/. The CHILDES Clinical English datasets are available at https://talkbank.org/childes/access/Clinical-Eng/. The DementiaBank datasets are available at https://talkbank.org/dementia/access/ and https://talkbank.org/dementia/ADReSS-2020/. Source data are provided with this paper.

## Code availability

All codes are available at Github.

## References

[1] Jones, C. H. & Dolsten, M. Healthcare on the brink: navigating the challenges of an aging society in the united states. *npj Aging* **10**, 22 (2024). 10.1038/s41514-024-00148-2 PMC10998868 38582901

[2] Thirunavukarasu, A. J. et al. Large language models in medicine. *Nat. Med.* **29**, 1930–1940 (2023). 10.1038/s41591-023-02448-8 37460753

[3] Xu, G. et al. A comprehensive survey of AI agents in healthcare. *J. Biomed. Informat.* **179**, 105045 (2026). 10.1016/j.jbi.2026.105045 PMC13435129 42009269

[4] National Institute on Deafness and Other Communication Disorders. Quick statistics about hearing, balance & dizziness. https://www.nidcd.nih.gov/health/statistics/quick-statistics-hearing (2020). Accessed: March 12, 2025.

[5] Notara, V., Vagka, E., Gnardellis, C. & Lagiou, A. The emerging phenomenon of nomophobia in young adults: a systematic review study. *Addiction health* **13**, 120 (2021). 10.22122/ahj.v13i2.309 PMC8519611 34703533

[6] Moyle, W. Grand challenge of maintaining meaningful communication in dementia care. *Front. Dement.* **2**, 1137897 (2023). 10.3389/frdem.2023.1137897 PMC11285537 39081994

[7] Murdoch, B. Privacy and artificial intelligence: challenges for protecting health information in a new era. *BMC Med. ethics* **22**, 1–5 (2021). 10.1186/s12910-021-00687-3 PMC8442400 34525993

[8] Zheng, X. et al. The interspeech 2025 speech accessibility project challenge. In Proc. Interspeech 2025, 3269–3273 (2025).

[9] Whisper ∣ openai research. Accessed: March 12, 2025.

[10] Google cloud speech-to-text. https://cloud.google.com/speech-to-text. Accessed: March 12, 2025.

[11] Asaithambi, S. et al. A secure and trustworthy blockchain-assisted edge computing architecture for industrial internet of things. *Sci. Rep.* **15**, 15410 (2025). 10.1038/s41598-025-00337-3 PMC12048497 40316586

[12] Qin, R. et al. Enabling on-device large language model personalization with self-supervised data selection and synthesis. In *Proceedings of the 61st ACM/IEEE Design Automation Conference*, 1–6 (2024).

[13] Shridhar, K., Stolfo, A. & Sachan, M. Distilling reasoning capabilities into smaller language models. *Findings of the Association for Computational Linguistics: ACL 2023* 7059–7073 (2023).

[14] Qin, R. et al. Empirical guidelines for deploying llms onto resource-constrained edge devices. *ACM Transactions on Design Automation of Electronic Systems* (2024).

[15] Rehabilitation, People With Aphasia After Stroke (RELEASE) Collaborators, R.: Predictors of poststroke aphasia recovery: A systematic review-informed individual participant data meta-analysis. *Stroke* **52**, 1778–1787 (2021). 10.1161/STROKEAHA.120.031162 PMC8078126 33719515

[16] Leonard, L. B. Specific language impairment across languages. *Speech and language impairments in children* 115–129 (2014).

[17] Tomblin, J. B. et al. Prevalence of specific language impairment in kindergarten children. *J. Speech, Lang., Hearing Res.* **40**, 1245–1260 (1997). 10.1044/jslhr.4006.1245 PMC5075245 9430746

[18] Kramarow, E. A. Diagnosed dementia in adults age 65 and older: United states, 2022. National Health Statistics Report 203, National Center for Health Statistics, CDC 10.15620/cdc/154766 (2024). 38912900

[19] Lin, C.-Y. Rouge: A package for automatic evaluation of summaries. In *Text Summarization Branches Out*, 74–81 (2004).

[20] Zhang, T., Kishore, V., Wu, F., Weinberger, K. Q. & Artzi, Y. Bertscore: Evaluating text generation with BERT. In Proc. 8th International Conference on Learning Representations (ICLR) (2020).

[21] Sellam, T., Das, D. & Parikh, A. P. Bleurt: Learning robust metrics for text generation. In Proc. 58th Annual Meeting of the Association for Computational Linguistics, 7881–7892 (2020).

[22] Koenecke, A. et al. Racial disparities in automated speech recognition. *Proc. Natl. Acad. Sci.* **117**, 7684–7689 (2020). 10.1073/pnas.1915768117 PMC7149386 32205437

[23] Holmlund, T. B. et al. Applying speech technologies to assess verbal memory in patients with serious mental illness. *npj Digital Med.* **3**, 33 (2020). 10.1038/s41746-020-0241-7 PMC7066153 32195368

[24] Cettolo, M., Bertoldi, N. & Federico, M. The repetition rate of text as a predictor of the effectiveness of machine translation adaptation. In *Proceedings of the 11th Conference of the Association for Machine Translation in the Americas: MT Researchers Track*, 166–179 (2014).

[25] Fraser, K. C. et al. Automated classification of primary progressive aphasia subtypes from narrative speech transcripts. *Cortex* **55**, 43–60 (2014). 10.1016/j.cortex.2012.12.006 23332818

[26] Huang, D., Nguyen, T.-S., Liausvia, F. & Wang, Z. Rap: A metric for balancing repetition and performance in open-source large language models. In Proceedings of the 2025 Conference of the Nations of the Americas Chapter of the Association for Computational Linguistics: Human Language Technologies (Volume 1: Long Papers), 1479–1496 (2025).

[27] Tweedie, F. J. & Baayen, R. H. How variable may a constant be? measures of lexical richness in perspective. *Computers Humanit.* **32**, 323–352 (1998).

[28] Covington, M. A. & McFall, J. D. Cutting the gordian knot: The moving-average type–token ratio (mattr). *J. Quant. Linguist.* **17**, 94–100 (2010).

[29] Jelinek, F., Mercer, R. L., Bahl, L. R. & Baker, J. K. Perplexity-a measure of the difficulty of speech recognition tasks. * J. Acoustical Soc. Am.* **62**, S63–S63 (1977).

[30] Radford, A. et al. Language models are unsupervised multitask learners. *OpenAI blog* **1**, 9 (2019).

[31] Rajkomar, A., Hardt, M., Howell, M. D., Corrado, G. & Chin, M. H. Ensuring fairness in machine learning to advance health equity. *Ann. Intern. Med.* **169**, 866–872 (2018). 10.7326/M18-1990 PMC6594166 30508424

[32] Xu, G. et al. Rethinking fairness in medical imaging: Maximizing group-specific performance with application to skin disease diagnosis. *Medical Image Anal.* 103950 (2026). 10.1016/j.media.2026.103950 41564634

[33] Berisha, V. & Liss, J. M. Responsible development of clinical speech AI: bridging the gap between clinical research and technology. *npj Digital Med.* **7**, 208 (2024). 10.1038/s41746-024-01199-1 PMC11316053 39122889

[34] Dankwa-Mullan, I. Health equity and ethical considerations in using artificial intelligence in public health and medicine. *Preventing chronic Dis.* **21**, E64 (2024). 10.5888/pcd21.240245 PMC11364282 39173183

[35] Mennella, C., Maniscalco, U., De Pietro, G. & Esposito, M. Ethical and regulatory challenges of ai technologies in healthcare: A narrative review. *Heliyon* **10** (2024). 10.1016/j.heliyon.2024.e26297 PMC10879008 38384518

[36] Tan, T. et al. Cba-whisper: Curriculum learning-based adalora fine-tuning on whisper for low-resource dysarthric speech recognition. In *Proc. Interspeech 2025*, 3309-3313 (2025).

[37] Müller-Eberstein, M., Yee, D., Yang, K., Mantena, G. V. & Lea, C. Hypernetworks for personalizing asr to atypical speech. *Trans. Assoc. Computational Linguist.* **12**, 1182–1196 (2024).

[38] Just, S. A. et al. Moving beyond word error rate to evaluate automatic speech recognition in clinical samples: Lessons from research into schizophrenia-spectrum disorders. *Psychiatry Research* 116690 (2025). 10.1016/j.psychres.2025.116690 40849998

[39] Zhong, T., Geng, M., Hu, S., Li, G. & Liu, X. Regularized federated learning for privacy-preserving dysarthric and elderly speech recognition. In Proc. Interspeech 2025, 2103–2107 (2025).

[40] Agarwal, D., Zhang, H., Yu, Y. & Wang, Q. State-of-the-art dysarthric speech recognition with metaicl for on-the-fly personalization. *arXiv preprint arXiv:2509.15516* (2025).

[41] MacWhinney, B. Understanding spoken language through talkbank. *Behav. Res. methods* **51**, 1919–1927 (2019). 10.3758/s13428-018-1174-9 PMC6546550 30511153

[42] NVIDIA. Canary-1b: A multilingual asr/ast model from nvidia nemo. https://huggingface.co/nvidia/canary-1b (2024).

[43] Radford, A. et al. Robust speech recognition via large-scale weak supervision. In *International conference on machine learning*, 28492–28518 (PMLR,2023).

[44] NVIDIA & Suno.ai. Parakeet-rnnt-1.1b: Fastconformer-transducer asr model from nvidia nemo & suno.ai. Hugging Face model card https://huggingface.co/nvidia/parakeet-rnnt-1.1b (2024).

[45] Baevski, A., Zhou, Y., Mohamed, A. & Auli, M. wav2vec 2.0: A framework for self-supervised learning of speech representations. *Adv. neural Inf. Process. Syst.* **33**, 12449–12460 (2020).

[46] Barrault, L. et al. Seamlessm4t: Massively multilingual & multimodal machine translation. *arXiv preprint arXiv:2308.11596* (2023).

[47] Frantar, E., Ashkboos, S., Hoefler, T. & Alistarh, D. Gptq: Accurate post-training quantization for generative pre-trained transformers. In Proc. 11th International Conference on Learning Representations (ICLR) (2023).

[48] Renze, M. The effect of sampling temperature on problem solving in large language models. In *Findings of the Association for Computational Linguistics: EMNLP 2024*, 7346–7356 (2024).

[49] Liu, Y. et al. On learning to summarize with large language models as references. In *Proceedings of the 2024 Conference of the North American Chapter of the Association for Computational Linguistics: Human Language Technologies (Volume 1: Long Papers)*, 8647–8664 (2024).

[50] Van Veen, D. et al. Adapted large language models can outperform medical experts in clinical text summarization. *Nat. Med.* **30**, 1134–1142 (2024). 10.1038/s41591-024-02855-5 PMC11479659 38413730

[51] Croxford, E. et al. Current and future state of evaluation of large language models for medical summarization tasks. *Npj health Syst.* **2**, 6 (2025). 10.1038/s44401-024-00011-2 PMC11928168 40124388

[52] Chen, Q. et al. Benchmarking large language models for biomedical natural language processing applications and recommendations. *Nat. Commun.* **16**, 3280 (2025). 10.1038/s41467-025-56989-2 PMC11972378 40188094

[53] Devlin, J., Chang, M.-W., Lee, K. & Toutanova, K. Bert: Pre-training of deep bidirectional transformers for language understanding. In *Proceedings of the 2019 conference of the North American chapter of the association for computational linguistics: human language technologies, volume 1 (long and short papers)*, 4171–4186 (2019).

[54] Brown, P. F., Pietra, V. J. D., Mercer, R. L., Pietra, S. A. D. & Lai, J. C. An estimate of an upper bound for the entropy of english. *Computational Linguist.* **18**, 31–40 (1992).

[55] Aboeitta, A., Sharshar, A., Nafea, Y. & Shehata, S. Bridging ASR and LLMs for dysarthric speech recognition: benchmarking self-supervised and generative approaches. In Proc. Interspeech 2025, 2123-2127 (2025).

[56] Phukon, B., Zheng, X. & Hasegawa-Johnson, M. Aligning ASR evaluation with human and LLM judgments: intelligibility metrics using phonetic, semantic, and NLI approaches. In Proc. Interspeech 2025, 5708–5712 (2025).

[57] Cheng, T.-H., Clemmensen, L. H. & Das, S. Beyond word error rate: Auditing the diversity tax in speech recognition through dataset cartography. *arXiv preprint arXiv:2603.05267* (2026).

