# Supplementary material for: Assessing the use of automatic speech recognition and large language models for individuals with language impairments
Source: Nat Commun. 2026 Sep 10;17:10716. doi: 10.1038/s41467-026-76677-z (PMC13658231; doi:10.1038/s41467-026-76677-z)
Supplement: Supplementary file 1 [file 41467_2026_76677_MOESM1_ESM.pdf]

Supplementary Information

Assessing the Use of Automatic Speech Recognition and Large Language Models for Individuals with Language Impairments

$$H_0 : P = C \quad \text{versus} \quad H_{a2} : C > P \tag{S1}$$

$$H_0 : P = C \quad \text{versus} \quad H_{a3} : P > C \tag{S2}$$

[1] GPTQ [1] is a widely used compression method, originally designed to compress Generative Pre-trained Transformer models (GPT) [2].

	Dataset	p-value (ROUGE) H_{a2}	p-value (ROUGE) H_{a3}	p-value (BERTSCORE) H_{a2}	p-value (BERTSCORE) H_{a3}	p-value (BLEURT) H_{a2}	p-value (BLEURT) H_{a3}
**Aphasia**	Baycrest	2.2e-02*	1.0e+00	1.3e-02*	1.0e+00	1.9e-02*	4.7e-01
	MSU	4.8e-03*	1.0e+00	1.4e-02*	1.0e+00	1.7e-02*	1.0e+00
	NEURAL	2.9e-08*	1.0e+00	1.8e-09*	1.0e+00	1.6e-05*	3.4e-01
	NEURAL_2	3.0e-05*	1.0e+00	1.4e-06*	9.7e-02	6.8e-04*	4.6e-03*
	UMD	4.2e-02*	1.0e+00	3.3e-01	1.0e+00	5.5e-01	1.7e-01
	UNH	5.1e-02	9.7e-04*	1.8e-02*	4.8e-04*	1.6e-04*	2.2e-04*
	Richardson	1.2e-08*	1.0e+00	2.8e-07*	1.0e+00	4.0e-05*	8.3e-03*
**LIC**	ENNI	2.4e-05*	1.0e+00	3.7e-06*	1.0e+00	2.4e-04*	1.0e+00
	EllisWeismer/30ec	5.8e-04*	1.0e+00	6.3e-05*	1.0e+00	2.5e-08*	1.0e+00
	EllisWeismer/30pc	5.3e-06*	1.0e+00	5.0e-07*	1.0e+00	1.6e-06*	1.6e-01
	EllisWeismer/42ec	4.6e-01	6.8e-01	4.8e-01	3.8e-01	2.8e-01	1.0e+00
	EllisWeismer/42pc	1.0e+00	2.9e-01	1.1e-01	1.5e-01	1.1e-01	4.2e-01
	EllisWeismer/54ec	1.0e+00	2.9e-01	1.0e+00	3.2e-02*	1.0e+00	1.0e+00
	EllisWeismer/54int	1.0e+00	1.0e+00	1.0e+00	1.0e+00	1.0e+00	4.9e-01
	EllisWeismer/66conv	1.0e+00	1.0e+00	1.0e+00	1.0e+00	1.0e+00	1.0e+00
	Poler	1.2e-01	4.8e-08*	4.7e-02*	5.6e-08*	3.4e-02*	3.2e-07*
**Dementia**	ADReSS-IS2020	8.7e-02	1.0e+00	2.5e-01	1.0e+00	9.9e-01	9.0e-01
	Delaware	1.0e+00	7.0e-01	7.2e-01	7.7e-01	1.0e+00	1.0e+00
	Lu	4.7e-03*	1.0e+00	1.5e-02*	1.0e+00	1.1e-01	1.0e+00
	Pitt/cookie	4.3e-12*	8.8e-02	2.0e-07*	1.0e+00	2.8e-02*	1.0e+00

Table S1: Bonferroni adjusted p-values from one-sided Wilcoxon tests on (S1) and (S2), with the alternative hypotheses being $H_{a2} : C > P$ and $H_{a3} : P > C$, respectively. p-values exceeding 1 after Bonferroni correction were capped at 1.

Table S2: Examples in UNH and Ellis/42ec dataset, illustrating how the ASR-LLM performance changed after removing identified issues in ASR-generated transcripts. Segments labelled [noise: ...] were identified as recognition noise and [rep: ...] as repetition; [...] indicates omitted transcript content. Values in the rightmost column are BLEURT or BERTScore, as indicated in each block header.

**UNH via Whisper-Medium + Gemma-2-9B-GPTQ**		
**Patient: 1209 tokens**		**BLEURT**
Original	So for this part, I want to ask you [...] [noise: MBC 뉴스 김수근입니다]. going to the... um... [rep: the... [...] the...] No. Yeah. [noise: 빨리] eating. Awesome.	-0.2599
**Control: 5109 tokens**		**BLEURT**
Original	Something, ... [noise: KATHRYN] The last time I had a really bad case of the flu was last year. [...] I knew him through [noise: ORP] you [...] [noise: amily] was only ever have like tiny dogs [rep: 아, [...] 아,] or just other [... 1013 Tokens ...]	-0.7138
Remove Repetition	Something, ... [noise: KATHRYN] The last time I had a really bad case of the flu was last year. [...] I knew him through [noise: ORP] you [...] [noise: amily] was only ever have like tiny dogs. or just other [... 1013 Tokens ...]	-0.4139
Remove Noise	Something, ... The last time I had a really bad case of the flu was last year. [...] I knew him through you [...] was only ever have like tiny dogs [rep: 아, [...] 아,] or just other [... 1013 Tokens ...]	-0.5601
Remove Both	Something, ... The last time I had a really bad case of the flu was last year. [...] I knew him through you [...] was only ever have like tiny dogs. or just other [... 1013 Tokens ...]	-0.3854
Truncate	Something, ... The last time I had a really bad case of the flu was last year. [...] I knew him through you [...] was only ever have like tiny dogs. or just other.	-0.3245
**Ellis/42ec via Whisper-Tiny + Gemma-7B-GPTQ**		
**Patient: 1501 tokens**		**BERTScore**
Original	It's too dangerous. [noise: Приходите, что это променное] Oh, maybe she can just sit here on these stairs. So that'd be our Yes. Oh, this. Okay. [noise: 안녕] Oh there. [rep: arA [...] arA] [...] Thank you. you're just gonna have to put	0.7949
Remove Repetition	It's too dangerous. [noise: Приходите, что это променное] Oh, maybe she can just sit here on these stairs. So that'd be our Yes. Oh, this. Okay. [noise: 안녕] Oh there. [...] Thank you. you're just gonna have to put	0.8020
Remove Noise	It's too dangerous. Oh, maybe she can just sit here on these stairs. So that'd be our Yes. Oh, this. Okay. Oh there. [rep: arA [...] arA] [...] Thank you. you're just gonna have to put	0.8204
Remove Both	It's too dangerous. Oh, maybe she can just sit here on these stairs. So that'd be our Yes. Oh, this. Okay. Oh there. [...] Thank you. you're just gonna have to put	0.8214
**Control: 1478 tokens**		**BERTScore**
Original	Thank you all the best. [noise: 我來的氣球] I have a lot of stuff in there, right? and sit in bed in each [noise: この後] I don't like dying hair. [noise: rashi fi kayfa alo] That's a good place for you. [rep: Oh, [...] oh,] [...] We're going to look at this.	0.7889
Remove Repetition	Thank you all the best. [noise: 我來的氣球] I have a lot of stuff in there, right? and sit in bed in each [noise: この後] I don't like dying hair. [noise: rashi fi kayfa alo] That's a good place for you. [...] We're going to look at this.	0.8043
Remove Noise	Thank you all the best. I have a lot of stuff in there, right? and sit in bed in each. I don't like dying hair. That's a good place for you. [rep: Oh, [...] oh,] [...] We're going to look at this.	0.8002
Remove Both	Thank you all the best. I have a lot of stuff in there, right? and sit in bed in each. I don't like dying hair. That's a good place for you. [...] We're going to look at this.	0.8017

Table S3: Dataset information, including the study purpose of the dataset and the sample size of the patient (P) and control (C)

[D] **Dataset**	**Study Purpose**	**P**	**C**
[D01] Baycrest	Given the Cinderella story book, the participants were asked to retell the story in their own words.	14	20
[D02] MSU	Ask participants about their experience of stroke.	16	18
[D03] NEURAL	Primarily interested in spoken language, especially discourse (connected speech).	48	48
[D04] NEURAL_2	Continuous investigations, same format as NEURAL.	74	42
[D05] UMD	This project compared standardized test and narrative language performance (AphasiaBank protocol).	18	16
[D06] UNH	Same as above.	35	12
[D07] Richardson	Same as above.	22	54
[D08] ENNI	The Edmonton Narrative Norms Instrument (ENNI) database was collected to develop measures and norms for storytelling using stimuli designed for the purpose.	44	125
[D09] Ellis/30ec	30-month child exposed to novel word learning tasks under varying processing demands	40	65
[D10] Ellis/30pc	30-month child interacting with parent during free play with toys	51	64
[D11] Ellis/42ec	42-month child interacting with examiner during semi-structured play with toys	41	43
[D12] Ellis/42pc	42-month child interacting with parent during free play with toys	39	29
[D13] Ellis/54ec	54-month child interacting with examiner during semi-structured play with toys	33	20
[D14] Ellis/54int	54-month child participating in a structured interview with examiner	31	36
[D15] Ellis/66conv	66-month child engaging in free conversation with examiner without toys	36	37
[D16] Poler	25 children with epilepsy (CWE) matched by age and gender to unaffected peers.	26	25
[D17] ADReSS -IS2020	"Cookie Theft" picture description task. Designed for classifying Alzheimer from normal.	54	54
[D18] Delaware	Picture descriptions and procedural narrative tasks (e.g., "Cookie Theft").	60	34
[D19] Lu	Cookie Theft picture descriptions.	28	26
[D20] Pitt/cookie	Cookie Theft picture descriptions.	306	242

Table S4: LLM Information

[L]**LLM**	**Weights (GB)**
[L01] Gemma-2-2B-GPTQ[1]	2.080
[L02] Gemma-2-2B	5.231
[L03] Gemma-2-9B-GPTQ	6.170
[L04] Gemma-7B-GPTQ	7.180
[L05] Gemma-7B	17.070
[L06] Gemma-2-9B	18.480
[L07] Ministral-8B-GPTQ	5.760
[L08] Mistral-7B-GPTQ	4.170
[L09] Ministral-3B	6.618
[L10] Mistral-7B	14.500
[L11] Ministral-8B	16.030
[L12] Phi-3.5-mini-GPTQ	2.280
[L13] Phi-3.5-mini	7.640
[L14] Llama-3.2-1B-GPTQ	1.520
[L15] Llama-3.2-3B-GPTQ	2.260
[L16] Llama-3.2-1B	2.470
[L17] Llama-2-7B-GPTQ	3.900
[L18] Llama-3.2-3B	6.430
[L19] Llama-2-7B	13.480
[L20] Qwen2-2B-GPTQ	2.480
[L21] Qwen2-2B	4.419
[L22] Qwen2-7B-GPTQ	6.920
[L23] Qwen2-7B	12.674

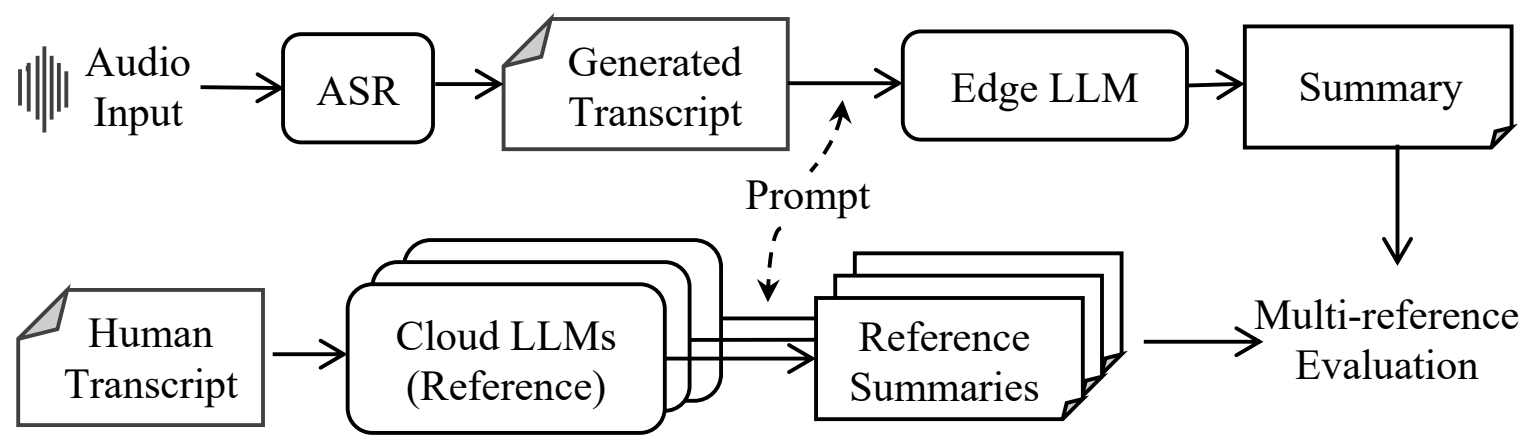

Figure S1: Overview of the ASR–LLM evaluation pipeline.

**Dataset**		**ROUGE** Max Std	**ROUGE** Max Range	**BERTScore** Max Std	**BERTScore** Max Range	**BLEURT** Max Std	**BLEURT** Max Range
Richardson	P	0.0473	0.1103	0.0010	0.0022	0.0447	0.1191
	C	0.0066	0.0148	0.0015	0.0038	0.0088	0.0247
EllisWeismer/66conv	P	0.0107	0.0304	0.0017	0.0041	0.0361	0.0958
	C	0.0102	0.0273	0.0019	0.0055	0.0208	0.0525
ADReSS-IS2020	P	0.0146	0.0460	0.0016	0.0047	0.0303	0.0742
	C	0.0151	0.0391	0.0037	0.0097	0.0268	0.0650

Table S5: Hyperparameter sensitivity analysis.

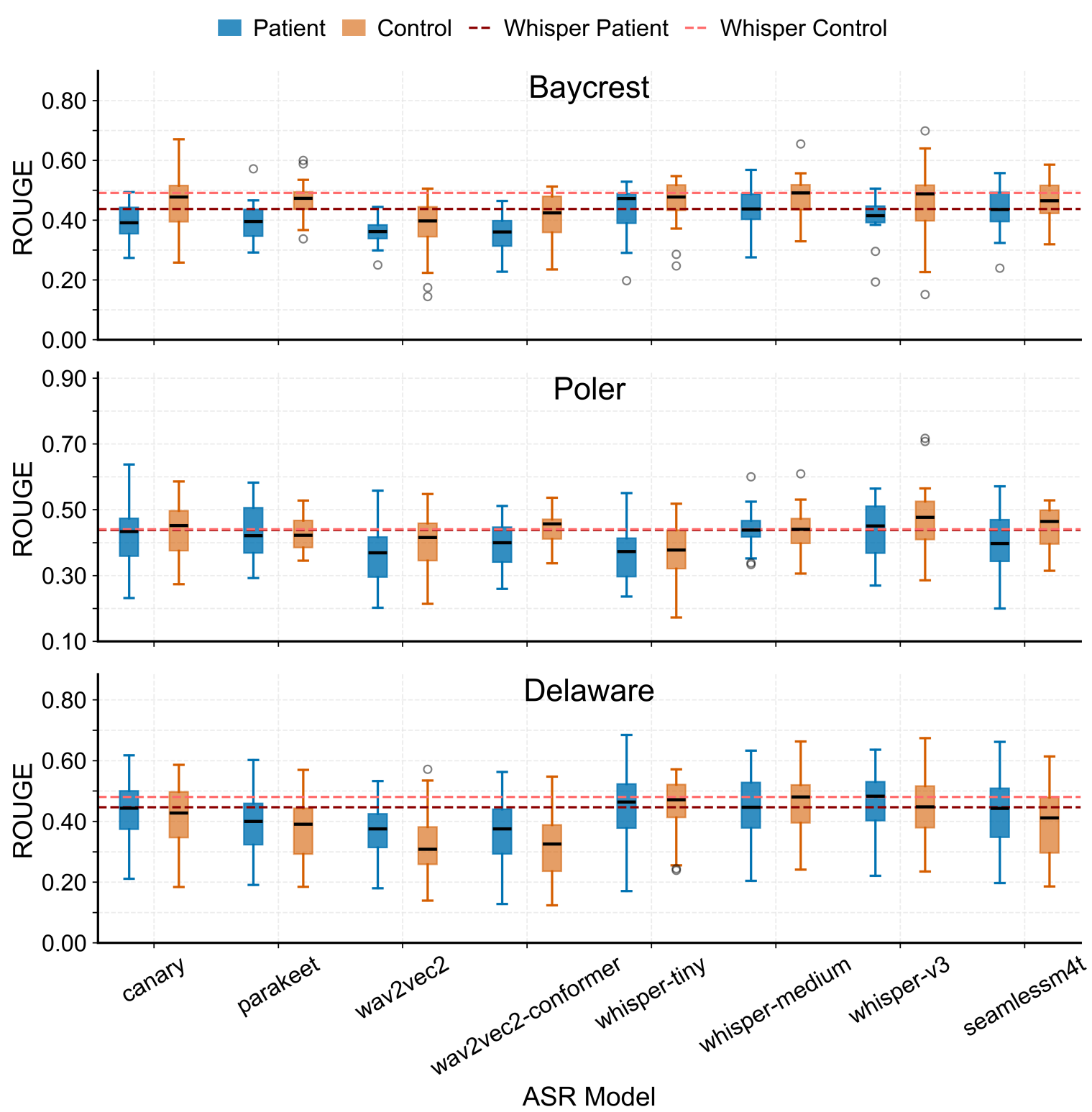

Figure S2: ROUGE-1 scores by ASR model for patient and control speakers. Each point is one participant. Whiskers extend to the most extreme points within 1.5 × IQR of the nearer quartile; points beyond the whiskers are outliers. Sample sizes: Baycrest, patient $n = 14$, control $n = 20$ for every ASR; Poler, patient $n = 26$ for every ASR, control $n = 26$ for canary, parakeet, wav2vec2, wav2vec2-conformer and seamless-m4t, and $n = 25$ for whisper-tiny, whisper-medium and whisper-large-v3; Delaware, patient $n = 60$, control $n = 34$ for every ASR.

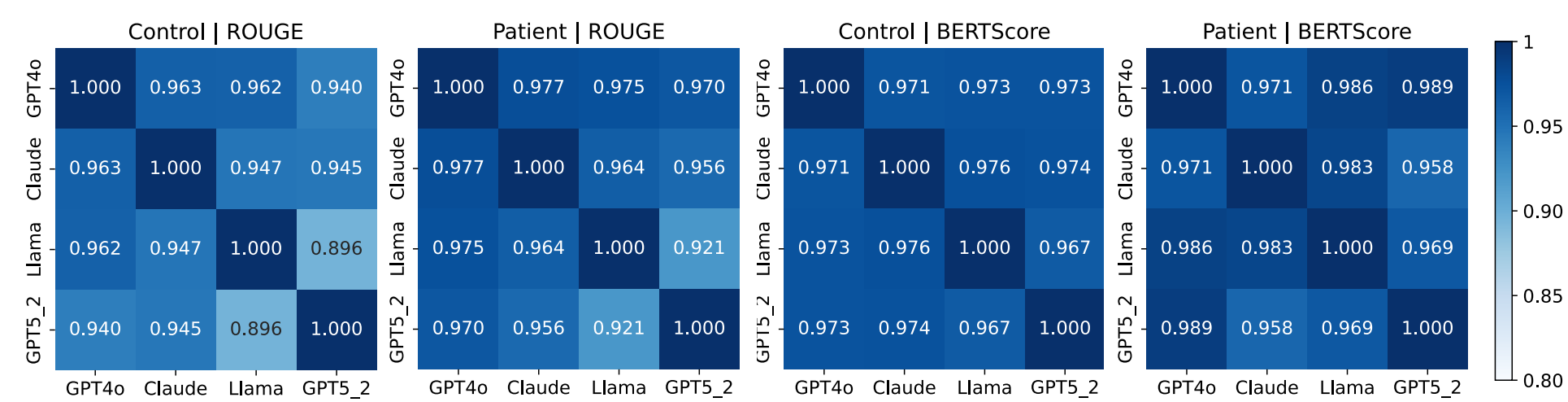

Figure S3: Agreement heatmaps of evaluation scores derived from different reference LLMs.

Supplementary References

[1] Elias Frantar, Saleh Ashkboos, Torsten Hoefler, and Dan Alistarh. Gptq: Accurate post-training quantization for generative pre-trained transformers. *arXiv preprint arXiv:2210.17323*, 2022.

[2] Luciano Floridi and Massimo Chiriatti. Gpt-3: Its nature, scope, limits, and consequences. *Minds and Machines*, 30:681–694, 2020.
