# Supplementary material for: Assessing the use of automatic speech recognition and large language models for individuals with language impairments
Source: Nat Commun. 2026 Sep 10;17:10716. doi: 10.1038/s41467-026-76677-z (PMC13658231; doi:10.1038/s41467-026-76677-z)
Supplement: Supplementary file 2 [file 41467_2026_76677_MOESM2_ESM.pdf]

nature portfolio

Peer Review File

Assessing the Use of Automatic Speech Recognition and Large Language Models for Individuals with Language Impairments

Corresponding Author: Professor Yiyu Shi

This file contains all reviewer reports in order by version, followed by all author rebuttals in order by version.

Version 0:

Reviewer comments:

Reviewer #1

(Remarks to the Author)
Review of: Serving Individuals with Language Impairments using Automatic Speech Recognition Models and Large Language Models: Challenges and Opportunities

Regarding the scope of the paper, the subject is very interesting, tackling the impaired population, suffering from aphasia and dementia.

The paper is generally well structured. however, some major improvements could be added.

1- A major problem, considering the goal is to assess the results for Edge computing, and the availability of such devices, a practical lab would have been of a great support for acceptance. Indeed, the authors report message like:
- CUDA out of memory.
- Model sizes were below 20 GB, allowing them to run on commonly available hardware such as NVIDIA Jetson Orin or NVIDIA Jetson AGX.
- For original LLMs, they were trained from scratch (no evidence of such development on the paper)
- The compression technique used GPTQ [42], a widely used LLM compression method.

There is no evidence that the authors run the experiments on a physical edge platform.

2- Using GPT-4o as the only evaluator for ground truth production is questionable:
a. The user should have used a known dataset with ground truth for evaluations.
b. For evaluation, no specification of the workstation used and the simulation deployment architecture.
c. To prove the Edge deployment feasibility, some of the metrics like, energy consumption, processing time.
3- The whisper+LLM processing need to be detailed and showcased.
4- The metrics used are confronted by the fact that GPT4o is considered the ‘ground truth’ of the audio files.

The papers methodology is set to test a very large model matrix, 5 whisper variants x 23 LLMs, which is ambitious; but suffers from deep conceptual issues:
- Considering gpt 4o as sole source of ground truth transcription, lack scientific rigor. Model on model bias cannot be ignored. The use of other state-of-the-art models could have brought some clarity, in the absence of real ground truth, like Claude or Gemini…
- Hyper parameters are mentioned but no methods or strategy for parameter selection is specified.
- Reporting a token count for errors is not rigorous, as it depends on the workstation used.

Overall, the paper is ambitious about assessing such a large matrix of cross models, but falls short in meeting scientific rigor, necessary from such works.

(Remarks on code availability)
Review of: Serving Individuals with Language Impairments using Automatic Speech Recognition Models and Large Language Models: Challenges and Opportunities

Regarding the scope of the paper, the subject is very interesting, tackling the impaired population, suffering from aphasia and dementia.

The paper is generally well structured. however, some major improvements could be added.

1- A major problem, considering the goal is to assess the results for Edge computing, and the availability of such devices, a practical lab would have been of a great support for acceptance. Indeed, the authors report message like:
- CUDA out of memory.
- Model sizes were below 20 GB, allowing them to run on commonly available hardware such as NVIDIA Jetson Orin or NVIDIA Jetson AGX.
- For original LLMs, they were trained from scratch (no evidence of such development on the paper)
- The compression technique used GPTQ [42], a widely used LLM compression method.

There is no evidence that the authors run the experiments on a physical edge platform.

2- Using GPT-4o as the only evaluator for ground truth production is questionable:
a. The user should have used a known dataset with ground truth for evaluations.
b. For evaluation, no specification of the workstation used and the simulation deployment architecture.
c. To prove the Edge deployment feasibility, some of the metrics like, energy consumption, processing time.
3- The whisper+LLM processing need to be detailed and showcased.
4- The metrics used are confronted by the fact that GPT4o is considered the ‘ground truth’ of the audio files.

The papers methodology is set to test a very large model matrix, 5 whisper variants x 23 LLMs, which is ambitious; but suffers from deep conceptual issues:
- Considering gpt 4o as sole source of ground truth transcription, lack scientific rigor. Model on model bias cannot be ignored. The use of other state-of-the-art models could have brought some clarity, in the absence of real ground truth, like Claude or Gemini…
- Hyper parameters are mentioned but no methods or strategy for parameter selection is specified.
- Reporting a token count for errors is not rigorous, as it depends on the workstation used.

Overall, the paper is ambitious about assessing such a large matrix of cross models, but falls short in meeting scientific rigor, necessary from such works.

Reviewer #2

(Remarks to the Author)
This study assesses ASR-LLM performance under three steps (feasibility, capability via text similarity, differences between patients and controls).

Strengths:
- Plenty of experiments conducted.
- Well-written and organized.

Weaknesses:
Although it is an interesting study and the first of its kind, I have some concerns:

- For ASR, the authors employ ASR models without further fine-tuning. Literature suggests that fine-tuning through Parameter Efficient Fine-Tuning (PEFT) approaches (e.g., LoRA, Adapters) is needed to improve ASR metrics.
- The authors do not cite prior works on ASR and PEFT. For instance, the authors could cite some works of the Speech Accessibility Challenge at INTERSPEECH 2025 (https://www.isca-archive.org/interspeech_2025/index.html)
- For evaluation metrics: Literature has suggested several evaluation metrics apart from WER. However, no literature is cited. Please, review for example the Speech Accessibility Challenge at INTERSPEECH 2025.
Why did you choose these evaluation metrics in your study?

- Why did you choose the prompt of summarization for LLMs? The motivation of this choice seems to be a bit confusing. Additionally, the authors should write this prompt into the main text (not only in the Appendix).

(Remarks on code availability)

Reviewer #3

(Remarks to the Author)
This manuscript presents a systematic evaluation of edge-based ASR-LLM (Automatic Speech Recognition combined with Large Language Models) systems for serving individuals with language impairments across three clinical populations: Aphasia, Language Impairments in Children (LIC), and Dementia. The authors tested 115 ASR-LLM combinations across 20 TalkBank datasets and identified key factors (repetition, noise, transcript length) affecting system performance. The work addresses an important and timely question regarding the inclusivity of AI-powered healthcare communication tools.
The use of existing metrics (ROUGE, BERTScore, BLEURT) in evaluating method and standard statistical tests (Wilcoxon with Bonferroni correction) is not much novel. The novelty appears incremental rather than transformative so the claim should be normalized in introduction. Currently, a huge debate is going for the use of AI or in your case the GPT-4o as judge, the output on human transcripts as the "reference" or "best case" raises significant methodological concerns. I believe the GPT-4o summaries are not ground truth; they represent one model's interpretation, is there any HITL ? This approach conflates ASR errors with LLM interpretation differences.The authors acknowledge this limitation implicitly but should provide stronger justification or alternative validation.I am bit confused on the identification of ERROR1 (CUDA out of memory) and ERROR2 (probability tensor errors) in the main paper,these are implementation artifacts rather than fundamental limitations. Modern solutions (gradient checkpointing, flash attention, context windowing) could mitigate these issues. The 6,000-token threshold identified is specific to the hardware/software configuration used. I also think that the interpretation of "significance" should be contextualized with clinical meaningfulness. Please discuss the ethical considerations for deploying potentially biased systems in vulnerable populations. The comparison of only four ASR models (Figure 9) to select Whisper seems limited. More recent models (e.g., Whisper v3, SeamlessM4T) should be discussed. Temperature=0.1 and top_p=0.9 were used for "stable generation," but no sensitivity analysis was performed on these hyperparameters. Methodology combines existing techniques; no new algorithms or fundamentally new insights. I suggest authors clarify their contribution in intro. The work may be more suitable for a specialized venue (e.g.,informatics alike) unless the authors can strengthen the novelty and broader impact claims substantially. I have seen code, did I miss scripts? it seems to me more results and visuals.

(Remarks on code availability)
yes, it seems more result rather scripts.

Version 1:

Reviewer comments:

Reviewer #1

(Remarks to the Author)
I have reviewed the authors’ responses to the comments. The authors have addressed the remarks appropriately, the quality has been enhanced, and the paper has been prepared more thoroughly. I am satisfied with their revisions and recommend the publication of this paper in the journal.

(Remarks on code availability)
The code analysis showed that all parts of the code were written in an understandable manner. The results of the study with their implementation workflow were completely understandable to me. The code lacked proper documentation which created difficulties in understanding certain implementation details.

The code remains functional for use and has the ability to produce identical results through different methods but better community access to the code would result from adding more explanatory content and commentary. The complete explanation of main functions together with model architecture and training pipeline documentation provides readers with the necessary information to understand and replicate the experiments.

The project requires a complete README file which should contain all essential information about how to install the software, which dependency versions to use, how to prepare datasets, and provide users with examples of how to execute the software.

Open Access This Peer Review File is licensed under a Creative Commons Attribution 4.0 International License, which permits use, sharing, adaptation, distribution and reproduction in any medium or format, as long as you give appropriate credit to the original author(s) and the source, provide a link to the Creative Commons license, and indicate if changes were made.
In cases where reviewers are anonymous, credit should be given to 'Anonymous Referee' and the source.
The images or other third party material in this Peer Review File are included in the article's Creative Commons license, unless indicated otherwise in a credit line to the material. If material is not included in the article's Creative Commons license and your intended use is not permitted by statutory regulation or exceeds the permitted use, you will need to obtain permission directly from the copyright holder.
To view a copy of this license, visit https://creativecommons.org/licenses/by/4.0/

Response to Reviewers

We sincerely thank all three reviewers for the time, care, and expertise they devoted to evaluating our manuscript. We appreciate the reviewers' recognition of the strengths of this study. Reviewer 1 noted the scale of large model matrix. Reviewer 2 highlighted the extensive experiments and the overall clarity and organization of the manuscript. Reviewer 3 recognized the work as a systematic evaluation across three clinical populations and as an important and timely study of the inclusivity of AI-powered healthcare communication tools.

We are also grateful for their thoughtful and constructive feedback, which has helped us improve both the rigor and clarity of the paper. We have carefully considered all comments and suggestions. Below, we provide a point-by-point response and describe the corresponding revisions made in the manuscript.

1 Response to Reviewer 1

1) Physical Edge Device Validation *A major problem, considering the goal is to assess the results for Edge computing, and the availability of such devices, a practical lab would have been of a great support for acceptance. Indeed, the authors report message like: CUDA out of memory. / Model sizes were below 20 GB, allowing them to run on commonly available hardware such as NVIDIA Jetson Orin or NVIDIA Jetson AGX. / There is no evidence that the authors run the experiments on a physical edge platform.*

REPLY: We thank the reviewer for emphasizing the importance of validating our findings on physical edge devices. In response, we conducted additional experiments on two NVIDIA Jetson AGX Orin 64GB platforms. Due to the large scale of our study (115 ASR–LLM combinations across multiple datasets), running the full matrix on-device was computationally infeasible. We therefore performed two sets of targeted on-device experiments.

- **Targeted feasibility checks across all combinations.** For each ASR–LLM combination, we randomly selected three samples and evaluated them on the AGX devices.
- **Long-context stress testing.** For each ASR–LLM combination, we additionally selected the three samples with the longest transcripts in order to probe high-load on-device behavior.

Using the same visualization format as in the original lower-memory setting (24GB), we further constructed an on-device error matrix for the AGX experiments based on the long-context stress-test subset. The result is shown in Figure 1. Under these long-context conditions, most combinations in the LIC and Dementia datasets completed successfully, whereas a subset of combinations in Aphasia triggered runtime failures, primarily "CUDA out-of-memory" and "probability tensor errors". All observed failures occurred in long-context samples, which indicates that deployment behavior is strongly influenced by transcript length and available hardware memory. The AGX Orin 64GB represents a relatively high-end edge platform. Even under this generous memory budget, long-context inference remained computationally demanding.

These additional experiments refine our interpretation of the original failure patterns. The observed errors should be understood as resource-dependent deployment boundaries shaped by hardware budget, input length, and runtime constraints, rather than as evidence of fundamental

infeasibility of edge deployment. We have revised the manuscript accordingly. We believe these additions strengthen the manuscript by grounding the deployment analysis in physical edge hardware and by clarifying how feasibility changes across hardware budgets.

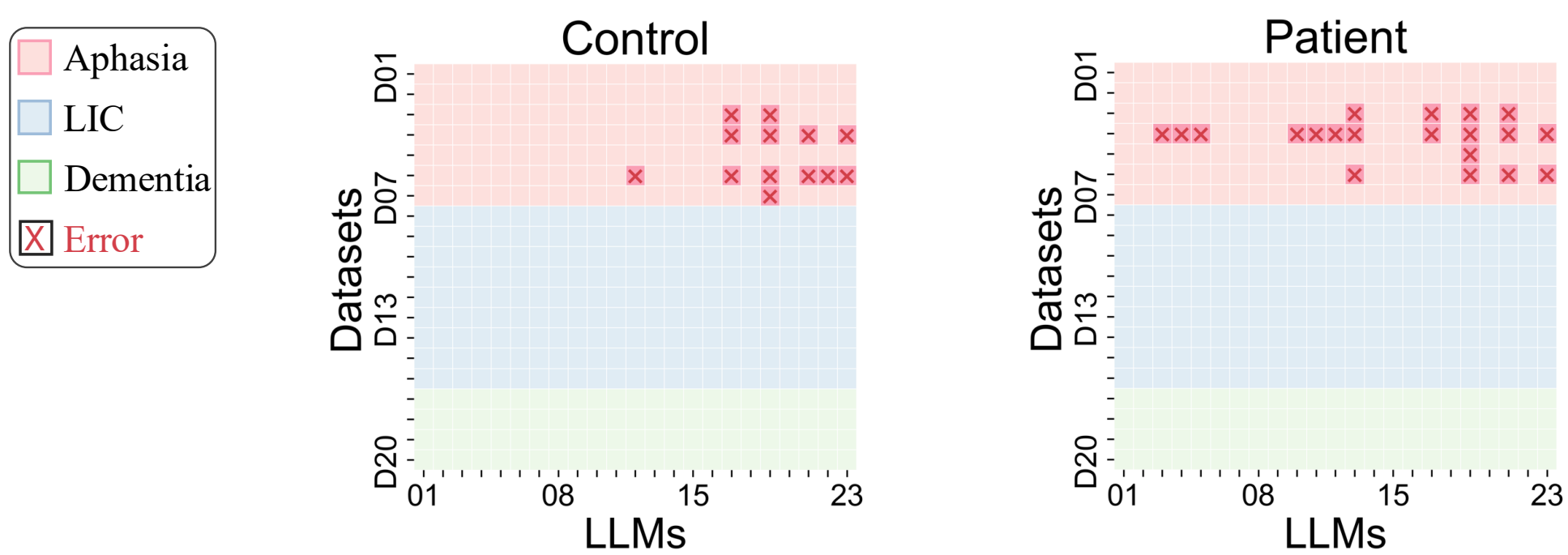

Figure 1: On-device stress-test matrix on the NVIDIA Jetson AGX platform. A grid is crossed if any selected long-context subset fails on that dataset.

The revised text now reads:

> "We begin with targeted experiments on physical edge hardware to examine how ASR–LLM systems behave under a high-memory on-device setting. These experiments were conducted on two NVIDIA Jetson AGX Orin 64GB platforms. Running the full 115-combination matrix on-device was computationally infeasible, so we designed a targeted evaluation strategy for each ASR–LLM combination. For each combination, we randomly selected three samples and also selected three the sample with the longest transcript. This design allowed us to cover both routine on-device execution and more demanding long-context cases without requiring a full on-device evaluation of the entire model matrix.
>
> Across these on-device experiments, all tested ASR models successfully processed the audio inputs and produced transcripts. Failures arose at the downstream LLM stage. In the LIC and Dementia datasets, all tested combinations completed inference successfully on the AGX platform. In Aphasia, failures were concentrated in several datasets, including NEURAL, NEURAL_2, UNH, Richardson, and UMD, across multiple LLMs. The observed failure types were "CUDA out-of-memory" and "Probability tensor contains either inf, 'nan' or element < 0". We note that these failure patterns are also influenced by implementation choices and may be mitigated in part by memory-efficient techniques such as flash attention, gradient checkpointing, or context windowing. At the same time, Aphasia transcripts were typically longer than those in the other two conditions, which suggests that input length plays an important role in determining whether the downstream LLM can complete inference on-device. These observations motivate a closer analysis of runtime, energy consumption, and memory usage on the edge platform. "

2) GPT-4o as Ground Truth *Using GPT-4o as the only evaluator for ground truth production is questionable. The user should have used a known dataset with ground truth for evaluations.*

The metrics used are confronted by the fact that GPT4o is considered the 'ground truth' of the audio files. Considering GPT-4o as sole source of ground truth transcription, lack scientific rigor. Model on model bias cannot be ignored. The use of other state-of-the-art models could have brought some clarity, in the absence of real ground truth, like Claude or Gemini.

REPLY: We thank the reviewer for highlighting this important concern. We agree that using GPT-4o alone as the reference could introduce model-specific bias. In response, we revised the evaluation protocol to use a multi-LLM reference based on four strong cloud models, Claude, GPT-4o, GPT-5.2, and Llama. For each sample, we generated reference summaries from the human-annotated transcript using all four reference models under the same prompt setting. We then computed ROUGE, BERTScore, and BLEURT between each ASR–LLM output and each reference summary separately, and averaged the resulting scores to obtain the final evaluation result. Using this multi-reference protocol, we recomputed Table 1, Table 2, and Table 3.

This revision led to three main observations. First, the overall performance values in Table 1 decreased slightly relative to the original GPT-4o-only evaluation, suggesting that a single GPT-4o reference may yield somewhat more favorable scores. Second, some localized changes appeared, most notably in BLEURT for the Aphasia datasets, where the relative pattern between patients and controls shifted in several cases. This supports the reviewer's concern that single-model references can introduce bias. Third, despite these local changes, the overall statistical patterns in Table 2 and Table 3 remained largely unchanged, indicating that the main conclusions of the study are robust to the choice of reference model.

To further examine potential model bias, we conducted a reference-level consistency analysis across the four reference LLMs. Specifically, we computed Pearson correlations between evaluation scores derived from Claude, GPT-4o, GPT-5.2, and Llama. We observed high agreement across reference models, with Pearson correlations mostly ranging from 0.92 to 0.98 across ROUGE, BERTScore, and BLEURT. As shown in Figure 2, this agreement is consistently strong for both patient and control groups.

Taken together, these additional analyses address the concern about model-on-model bias while showing that the main findings of the manuscript remain stable under a multi-reference evaluation setting. We have revised the manuscript accordingly.

The revised text now reads:

> " To reduce dependence on any single reference model, we construct a multi-LLM reference using four strong cloud-based LLMs, Claude, GPT-4o, GPT-5.2, and Llama. For each sample, each reference model generates a summary from the human transcript using the same prompt setting. We then compute ROUGE, BERTScore, and BLEURT between the ASR–LLM summary and each reference summary separately, and average the resulting scores to obtain the final evaluation score. Higher similarity indicates better ASR–LLM performance, as it suggests that the ASR–LLM output remains closer to reference summaries derived from the human transcript. "

> "To further examine the robustness of the multi-reference protocol, we computed pair-wise correlations between evaluation scores derived from different reference models. As shown in Figure 2, agreement across reference models is consistently high, with Pearson correlations ranging from approximately 0.89 to 0.98 across ROUGE and 0.95 to 0.99 across BERTScore for both patient and control groups. This indicates that evaluation outcomes are not overly dependent on any single reference model and supports the use of the aggregated multi-LLM reference in the main analysis."

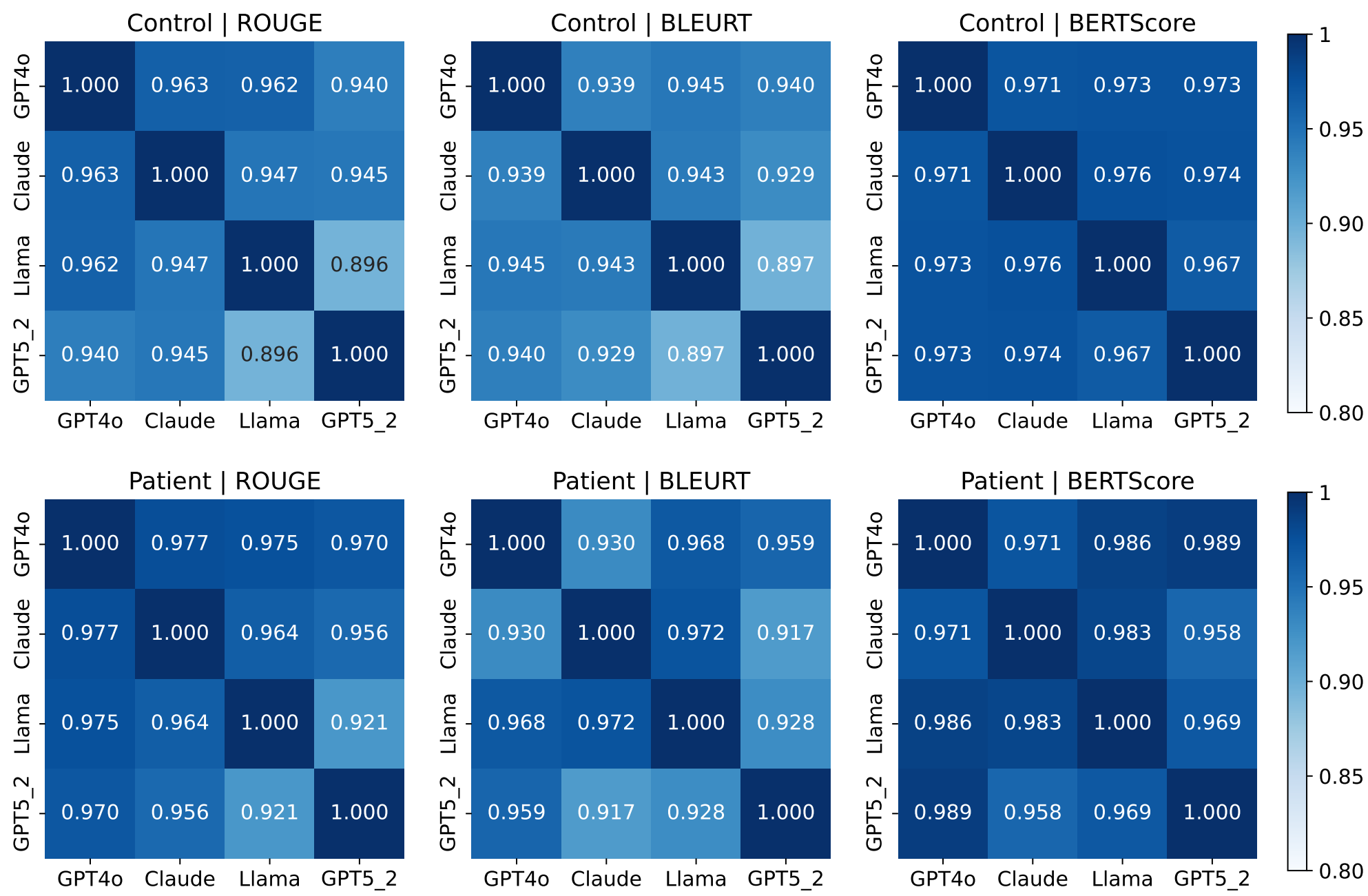

Figure 2: Agreement heatmaps of evaluation scores derived from reference LLMs for control and patient groups. High pairwise correlations indicate strong consistency across reference models.

3) Workstation Specifications and Deployment Architecture *For evaluation, no specification of the workstation used and the simulation deployment architecture.*
REPLY: We thank the reviewer for raising this important point. We agree that the original manuscript did not describe the experimental hardware settings clearly enough. In the revised manuscript, we have added a dedicated Methods subsection that specifies both the edge platform used for on-device validation and the server platform used for the large-scale full-matrix evaluation.

Edge setting. Targeted on-device validation was conducted on two NVIDIA Jetson AGX Orin developer kits, each equipped with 64GB of unified memory. These experiments were used to assess practical deployment behavior on physical edge hardware, including feasibility, latency, energy consumption, and memory usage.

Server setting. The full-matrix evaluation across 115 ASR–LLM combinations was conducted on the GPU cluster, with each job allocated a single NVIDIA A10 GPU with 24GB VRAM. This setting provided a standardized and more constrained memory budget for large-scale screening across datasets and model combinations, and it is the setting in which the reported ERROR1/ERROR2 patterns were observed.

Software environment. All experiments were implemented in Python using PyTorch and the Hugging Face ecosystem. Exact environment specifications and scripts are provided with the released code to support reproducibility.

We have revised the manuscript accordingly so that the hardware settings and their respective roles in the study are now stated explicitly.

The revised text now reads:

> " This study used two complementary hardware settings. Targeted on-device validation was first conducted on two NVIDIA Jetson AGX Orin developer kits, each equipped

with 64GB of unified memory. These edge experiments were designed to assess practical deployment behavior under a high-memory edge configuration, including inference feasibility, latency, energy consumption, and memory usage.

To complement the on-device analysis, the full-matrix evaluation across 115 ASR–LLM combinations was conducted on the GPU cluster, with each job allocated a single NVIDIA A10 GPU with 24GB VRAM. This configuration provided a standardized and more constrained memory budget for large-scale screening across datasets and model combinations.

All experiments were implemented in Python using PyTorch and the Hugging Face ecosystem. ”

4) Edge Deployment Feasibility Metrics *To prove the Edge deployment feasibility, some of the metrics like, energy consumption, processing time.*

REPLY: We thank the reviewer for this important suggestion. We agree that edge deployment feasibility should be assessed not only by whether inference can be completed on-device, but also by practical runtime metrics such as processing latency and energy consumption. In response, we added on-device latency and energy measurements on two NVIDIA Jetson AGX Orin 64GB platforms. In the revised manuscript, we now report these deployment metrics for both stages of the ASR–LLM pipeline. For the ASR stage, we measured latency and energy consumption as a function of audio duration across Whisper model sizes, as shown in Figure 3. For the downstream LLM stage, we measured latency and energy consumption as a function of transcript length across representative LLM size groups, as shown in Figure 4.

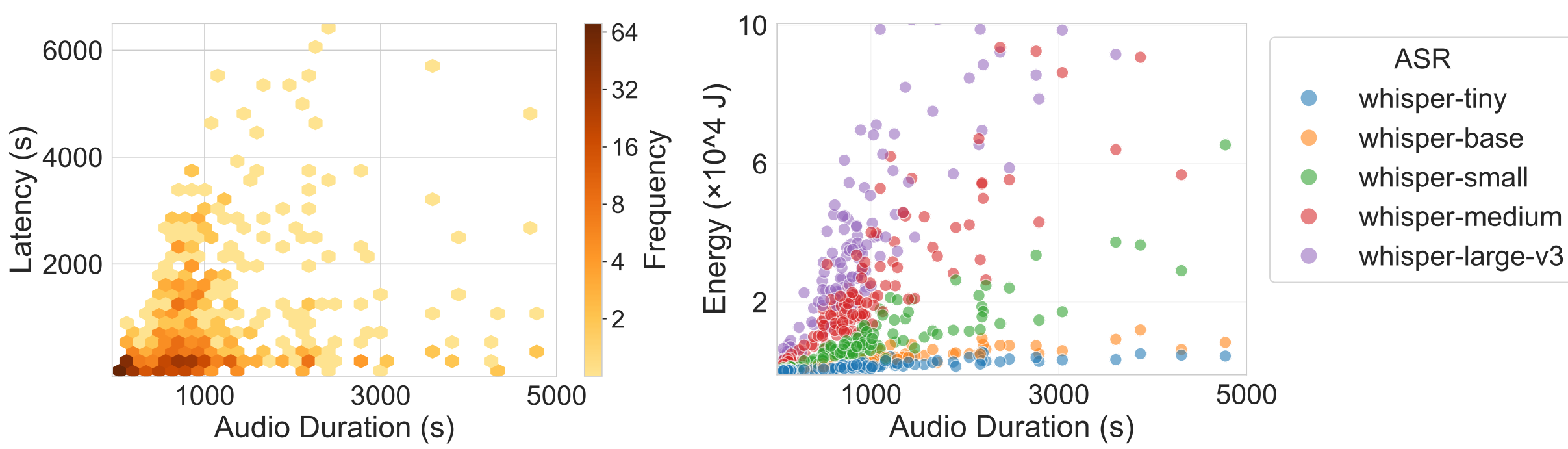

Figure 3: Latency and energy measurements for ASR stage on the edge.

The added experiments show that both stages become increasingly expensive as input size grows. ASR latency and energy consumption increase with audio duration, with larger Whisper models incurring higher energy cost. Similarly, LLM latency and energy consumption increase with transcript length, and long transcripts can require several minutes of on-device inference for a single sample. Larger LLMs also consume substantially more energy than smaller ones under comparable input lengths.

These results show that practical edge deployment is constrained by both processing time and energy consumption across the full ASR–LLM pipeline. Even on a high-memory edge device, long recordings and long transcripts remain costly to process. We have revised the manuscript

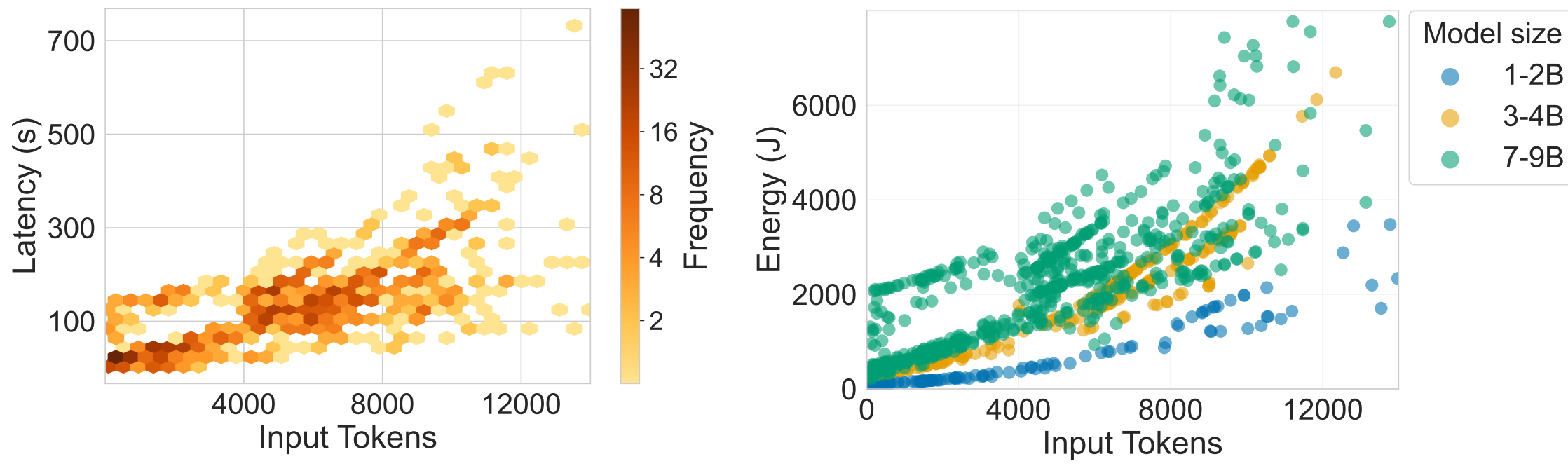

Figure 4: Latency and energy measurements for LLM stage on the edge.

accordingly to clarify that edge feasibility depends not only on whether a system can run on-device, but also on whether it can do so within practical deployment constraints.

The revised text now reads:

> " We next quantify the practical cost of on-device execution on the AGX platform for both stages of the ASR–LLM pipeline. As shown in Figure 3, the ASR stage becomes increasingly expensive as audio duration grows. ASR latency rises substantially for longer recordings, and the corresponding energy consumption also increases. In addition, ASR energy cost depends strongly on model size, with larger Whisper variants consuming substantially more energy than smaller ones for comparable audio durations. On the AGX platform, the energy required for audio transcription was often substantial. For Whisper-tiny and Whisper-base, the total energy consumption was generally below 10,000 J, whereas Whisper-medium and Whisper-large-v3 could easily exceeded 20,000 J. These results show that transcription itself already imposes a significant deployment cost on edge hardware.
>
> The downstream LLM stage shows a similar pattern with respect to transcript length, as shown in Figure 4. Latency increases markedly with input tokens, and long transcripts can require several minutes of inference for a single sample. Energy consumption also grows with input length and is strongly associated with LLM model size. Under comparable input lengths, larger models consistently consume more energy than smaller ones. For example, when the input length is around 4,000 tokens, models in the 1B–2B range typically consume less than 500J, whereas models in the 7B–9B range often consume more than 2,000J. These results show that practical deployment cost accumulates across both stages of the pipeline. Audio duration drives the cost of ASR transcription, while transcript length drives the cost of downstream summarization. Even under a high-memory edge setting using the 64GB NVIDIA Jetson AGX Orin platform, long recordings remain expensive in latency and energy. This motivates the next stage of analysis, in which we perform a full-matrix screening study under a lower and more standardized memory budget. "

5) ASR+LLM Pipeline Details *The whisper+LLM processing need to be detailed and showcased.*

REPLY: We thank the reviewer for this helpful suggestion. We agree that the original manuscript did not describe the end-to-end ASR–LLM pipeline clearly enough. In the revised manuscript, we have added both a dedicated pipeline figure (Figure 5) and a corresponding Methods description to make the processing flow more explicit.

Overview of the ASR–LLM pipeline. The main pipeline consists of two sequential stages. First, each audio recording is segmented using the timestamp annotations and transcribed by Whisper. The segment-level transcriptions are then concatenated in temporal order to form a complete per-participant transcript. We evaluate multiple Whisper model sizes to study how ASR quality affects downstream performance. Second, the ASR-generated transcript is passed to the downstream LLM using a standardized summarization prompt. All ASR–LLM combinations use the same prompt and decoding configuration to ensure a fair comparison.

Reference generation and evaluation. In parallel, the human-annotated transcript is provided to multiple reference LLMs using the same prompt template. These models generate reference summaries, which are then compared with the ASR–LLM summary using ROUGE, BERTScore, and BLEURT. The resulting scores are aggregated across reference models to obtain the final multi-reference evaluation score. This design isolates ASR-induced variation to the ASR–LLM pipeline while keeping the evaluation protocol consistent.

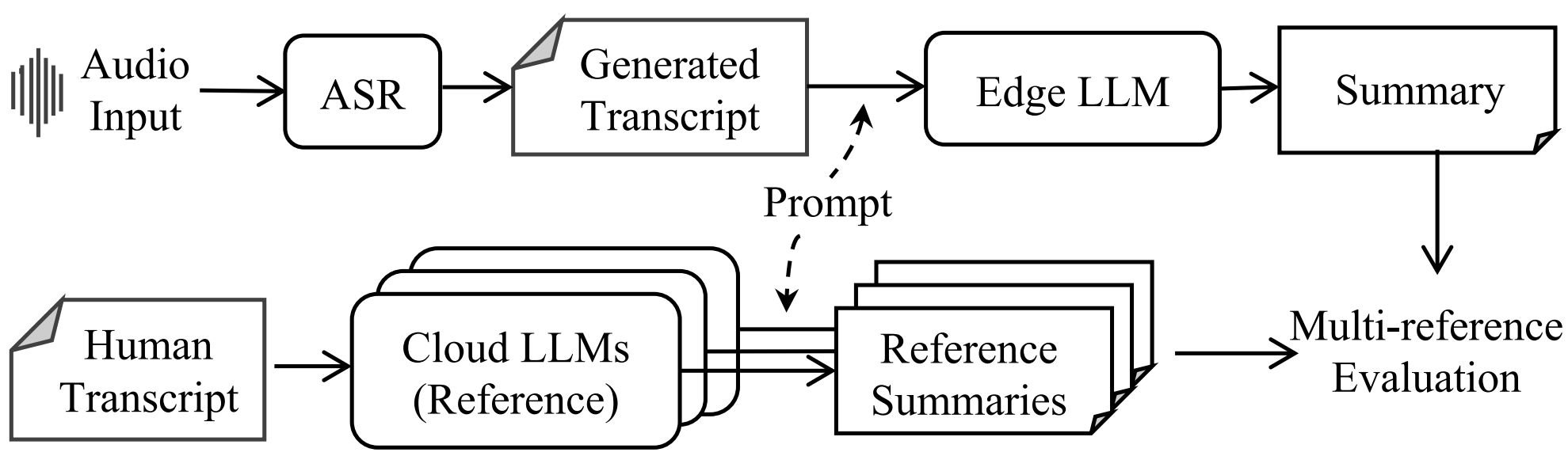

Figure 5: Overview of the ASR–LLM evaluation pipeline.

We have revised the manuscript accordingly so that the pipeline structure, reference generation process, and evaluation flow are now described explicitly and illustrated visually.

The revised text now reads:

> “ As illustrated in Figure 5, the ASR–LLM evaluation framework consists of two parallel paths. In the main pipeline, audio is first transcribed by the ASR model and the generated transcript is then summarized by the downstream edge LLM. In the reference pipeline, the human-annotated transcript is summarized by multiple cloud-based reference LLMs using the same prompt template. ROUGE, BERTScore, and BLEURT are computed between the ASR–LLM summary and each reference summary, and the resulting scores are aggregated to obtain the final evaluation score. ”

6) Hyperparameter Selection Strategy *Hyper parameters are mentioned but no methods or strategy for parameter selection is specified.*

REPLY: We thank the reviewer for this important comment. We agree that the original manuscript did not clearly explain the rationale behind the parameter choices. In the revised manuscript, we have added a dedicated Methods description of the parameter selection strategy.

Our parameter settings were guided by three principles, stable and reproducible generation, fair comparison across ASR–LLM combinations, and practical constraints of edge-oriented deployment.

For **model- and data-dependent settings**, the ASR sampling rate was fixed at 16,000 Hz following Whisper's pretraining configuration. We also imposed no input length limit on transcripts, in order to preserve full conversational context. This avoids truncating clinically meaningful content and prevents introducing bias in patient–control comparisons.

For **generation**, we used $temperature = 0.1$, $top_p = 0.9$, $top_k = 10$, and $do\_sample =$ `True`. This configuration was chosen to encourage stable and near-deterministic outputs while avoiding repetition artifacts that can arise under purely greedy decoding, especially for long transcripts. The output length was capped at 100 tokens to produce concise summaries while keeping generation cost bounded.

For **deployment-related settings**, compressed models used GPTQ 4-bit quantization with FP16 activations as a practical memory–quality trade-off. Batch size was set to 16 in the server-based full-matrix experiments and reduced to 1 for on-device evaluation on the Jetson AGX platform due to memory constraints.

To validate that these choices did not unduly affect the conclusions, we further conducted a sensitivity analysis over multiple $temperature/top_p$ settings, as reported in Table 1. Across representative models and datasets, BERTScore remained highly stable, while ROUGE and BLEURT showed only modest variation under more extreme settings. These results indicate that the selected configuration provides a conservative and robust setting for stable generation.

We have revised the manuscript accordingly. The revised text now reads:

> " For the default settings used to construct the ASR–LLM pipeline, we applied a unified configuration across all experiments to ensure fair comparison across model combinations, and compatibility with edge-oriented deployment constraints. The ASR sampling rate was fixed at 16,000 Hz, following Whisper's pretraining configuration [20]. For the LLM, no input length limit was imposed in order to preserve the full conversational context of each transcript, since truncation could remove clinically meaningful information and introduce bias in patient–control comparisons. The LLM output was capped at 100 tokens to produce concise summaries while keeping generation cost bounded.
>
> For decoding, we used $do\_sample =$ `True`, $temperature = 0.1$, $top_p = 0.9$, and $top_k = 10$. This configuration was chosen to encourage stable and near-deterministic generation while avoiding repetition artifacts that can arise under purely greedy decoding, especially for long transcripts[22]. For compressed models, we used GPTQ 4-bit quantization with FP16 activations as a practical memory–quality trade-off for edge-oriented inference. Batch size was set to 16 in the server-based large-scale experiments and reduced to 1 for on-device evaluation on the Jetson AGX platform due to memory constraints.
>
> To verify that the decoding configuration did not unduly affect the main conclusions, we further conducted a sensitivity analysis over multiple $temperature/top_p$ settings, as reported in Table 1. Across representative models and datasets from all three disease categories, BERTScore remained highly stable, with maximum range ≤ 0.01, while ROUGE and BLEURT showed only modest variation under more extreme settings. These results indicate that the selected configuration provides a conservative and robust setting for stable generation. "

7) Token-based Error *Reporting a token count for errors is not rigorous, as it depends on the workstation used.*

REPLY: We thank the reviewer for this important methodological observation. We agree that absolute token counts associated with failures are hardware-dependent and should not be interpreted as universal thresholds. We have revised the manuscript accordingly.

In the revised version, we no longer present token counts as fixed error limits or emphasize specific token thresholds in the main text. Instead, we focus on the broader trend that failure likelihood increases with input length. To support this more general interpretation, we added a binned analysis of error rate as a function of input tokens, shown in Figure 6. This analysis shows a clear monotonic increase in error rate for longer inputs and is intended to characterize the trend in a way that is less dependent on any single workstation configuration. We also revised the corresponding Results text so that transcript length is discussed as a resource-dependent factor associated with downstream failures, without implying a universal cutoff. This shifts the interpretation from token-specific thresholds to deployment-relevant failure patterns under constrained hardware settings.

These revisions improve the rigor of the presentation and better align the analysis with the reviewer's concern.

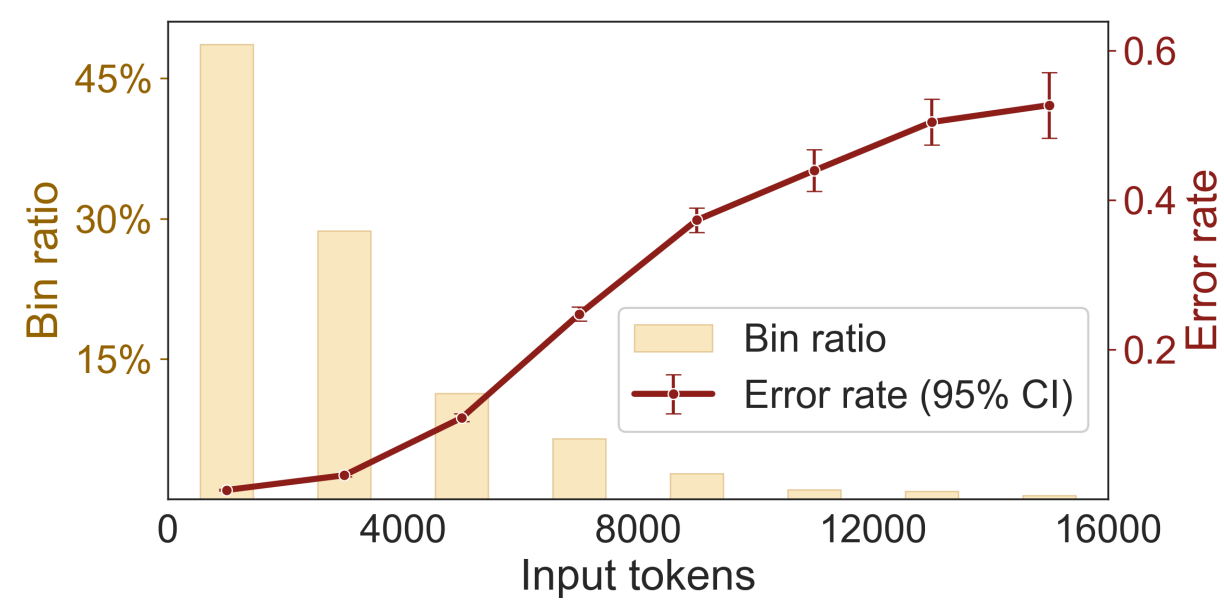

Figure 6: Input-length distribution and corresponding error rate across token bins.

The revised text now reads:

> " Transcript length showed a strong association with downstream failure behavior. The left panel presents a representative example in which memory demand increases with input length. The middle panel provides a schematic view of how long-context inference can place increasing pressure on the downstream LLM runtime. The right panel shows the population-level trend across token-length bins, where error rates increase monotonically for longer inputs. We note that these observed failure patterns are also influenced by implementation choices, and that memory-efficient techniques such as flash attention, gradient checkpointing, or context windowing may shift the practical boundary at which they arise. Even with this caveat, the broader trend is consistent. Longer transcripts place greater memory and numerical demands on downstream LLM inference and are therefore more likely to trigger failures under constrained deployment settings. "

2 Response to Reviewer 2

1) PEFT for Better Performance *For ASR, the authors employ ASR models without further fine-tuning. Literature suggests that fine-tuning through Parameter Efficient Fine-Tuning (PEFT) approaches (e.g., LoRA, Adapters) is needed to improve ASR metrics.*

REPLY: We thank the reviewer for this important suggestion. We agree that parameter-efficient fine-tuning methods such as LoRA and adapters are promising for improving ASR performance, especially for atypical or impaired speech. We also agree that adaptation is likely to be an important direction for reducing the performance gaps observed in this study.

Our current work, however, focuses on evaluating off-the-shelf ASR–LLM systems under edge-oriented deployment constraints, rather than on model adaptation. We chose this scope intentionally in order to assess how existing edge-deployable systems behave before any task-specific personalization or fine-tuning is applied.

We did not include PEFT-based ASR adaptation in the present study for three practical reasons. First, on-device inference was already computationally demanding even on the NVIDIA Jetson AGX Orin 64GB, which represents a relatively high-end edge platform. As shown in Figure 3 and Figure 4, both ASR transcription and downstream LLM summarization incurred substantial latency and energy cost on-device, with long recordings and long transcripts often requiring several minutes to process. These results suggest that adding on-device training or PEFT would introduce substantial additional demands beyond the current deployment budget. Second, realistic edge settings typically provide only limited user-specific speech data, making stable adaptation difficult in practice. Third, many effective fine-tuning workflows rely on pooling data across users, which introduces tension with the privacy-first motivation for local clinical speech processing.

We therefore view PEFT-based adaptation as an important limitation and future direction. In the revised manuscript, we now state this explicitly in the Discussion. We also note several promising directions for future work, including PEFT-based ASR adaptation, few-shot or in-context personalization, and privacy-preserving distributed training approaches such as federated learning. These methods may help improve robustness for users with language impairments while remaining compatible with the practical constraints of edge deployment.

The revised text now reads:

> " The performance gaps observed in this study suggest that user-specific adaptation may be an important direction for improving ASR–LLM systems, especially for individuals with language impairments. Speech patterns in these populations are highly heterogeneous, and a pipeline tailored to a user's linguistic profile may improve both transcription quality and downstream summarization. In this sense, personalization may be particularly beneficial for the groups that are currently the most difficult to serve reliably.
>
> At the same time, personalization remains challenging under the present edge deployment setting. Our edge experiments show that long-context inference already places substantial demands on memory, latency, and energy. This makes additional adaptation steps difficult to support on current hardware. A second challenge is data availability. In realistic edge settings, a device may observe only a limited amount of user-specific speech, which makes continual learning and stable adaptation difficult in practice.
>
> For these reasons, the most suitable form of personalization remains an open ques-

tion. PEFT-based methods such as LoRA or adapters are promising directions [16, 23], particularly when larger curated datasets or privacy-preserving training strategies are available [13, 28]. Few-shot and in-context adaptation are also appealing because they may allow limited personalization without modifying model parameters [2]. Future work should examine which adaptation strategies can most effectively improve robustness for language-impaired users while remaining compatible with the practical constraints of edge deployment. Broader benchmark resources that better represent diverse language impairment profiles will also be important for evaluating these approaches under clinically relevant conditions [5]. ”

“In addition, the present study evaluates off-the-shelf systems under edge-oriented deployment constraints and does not include task-specific adaptation or personalization. A further limitation concerns the interpretation of statistical significance.”

2) Prior Work Citations *The authors do not cite prior works on ASR and PEFT. For instance, the authors could cite some works of the Speech Accessibility Challenge at INTERSPEECH 2025 (link)*

REPLY: We thank the reviewer for pointing us to the Speech Accessibility Challenge at INTERSPEECH 2025 and the broader literature on ASR for individuals with speech disabilities. We agree that the original manuscript did not cite this prior work adequately. In the revised manuscript, we have added citations to relevant benchmark, PEFT, and personalization studies and now discuss them explicitly in the related work and discussion sections.

In particular, we now cite the INTERSPEECH 2025 Speech Accessibility Challenge overview [27] as an important benchmark for impaired-speech ASR, and CBA-Whisper [23] as an example of PEFT-based adaptation using AdaLoRA for dysarthric speech recognition. We also added citations to recent work on lightweight personalization without parameter updates [2] and privacy-preserving distributed adaptation [28], as these are relevant future directions for edge-oriented deployment.

These additions help position our work more clearly with respect to the current literature. In particular, they clarify that our study focuses on evaluating off-the-shelf ASR–LLM behavior under edge deployment constraints, while PEFT-based adaptation and personalization remain important complementary directions for future work.

3) Evaluation Metrics *For evaluation metrics: Literature has suggested several evaluation metrics apart from WER. However, no literature is cited. Please, review for example the Speech Accessibility Challenge at INTERSPEECH 2025. Why did you choose these evaluation metrics in your study?*

REPLY: We thank the reviewer for raising this important point and for directing us to the Speech Accessibility Challenge at INTERSPEECH 2025. We agree that the original manuscript did not sufficiently justify the evaluation metrics or situate them within the broader literature. In response, we carefully reviewed the relevant work on impaired-speech ASR evaluation and have revised the manuscript to include both additional citations and a clearer explanation of our metric choices.

Our study involves two distinct evaluation objectives, which require different metric suites. First, for ASR-generated transcripts, we use WER, PPL, TTR, and RR to assess transcript quality as an intermediate representation for the downstream LLM. WER measures recognition accuracy against the human transcript, while PPL, TTR, and RR capture fluency, lexical diversity, and

repetition patterns that are especially relevant in language-impaired speech. We selected these metrics because our goal is not to evaluate ASR accuracy in isolation, but to characterize transcript properties that may affect downstream LLM behavior. This framing is consistent with recent work arguing that WER alone is insufficient for evaluating ASR in clinical and atypical speech settings [13].

Second, for LLM-generated summaries, we use ROUGE, BERTScore, and BLEURT because they provide complementary views of output quality, covering lexical overlap, semantic similarity, and learned human-aligned quality. In revising the manuscript, we also reviewed the INTERSPEECH 2025 Speech Accessibility Challenge [27], whose evaluation philosophy similarly emphasizes semantic quality beyond word-level matching. We now cite this benchmark explicitly and clarify how our use of BERTScore and BLEURT aligns with this broader direction, while ROUGE provides an additional interpretable surface-level measure.

We also acknowledge that the literature has proposed several other evaluation metrics beyond WER, including CER, phonetic similarity measures, semantic robustness metrics, and downstream task-oriented evaluation protocols [1, 8, 19, 26]. We did not adopt these alternatives here because our focus is the effect of transcript quality on downstream ASR–LLM performance, and because the TalkBank datasets do not provide the additional task-specific annotations required by some of these methods. Nonetheless, we now cite these works and acknowledge them as important complementary directions in the revised manuscript.

Overall, we have revised the manuscript to make the rationale for both transcript-level and summary-level evaluation more explicit, and to better position our metric choices within the current literature on impaired-speech ASR and downstream evaluation.

The revised text now reads:

> " To characterize transcript quality, we use four complementary metrics that capture different aspects of the ASR output as an intermediate representation. Word Error Rate (WER) measures transcription accuracy against the human-annotated transcript. Perplexity (PPL) measures how well a language model predicts a given text, with lower values indicating more fluent and predictable language [6]. Type-Token Ratio (TTR), defined as the ratio of unique words to total words [9], measures lexical diversity and can reflect constrained language use or word-finding difficulty. Repetition Ratio (RR) quantifies the proportion of repeated words in the transcript [12], capturing perseveration and other repetition-related irregularities.
>
> We selected these metrics because they assess transcript quality beyond recognition accuracy alone. WER captures whether the ASR system recognizes the spoken words correctly, whereas PPL, TTR, and RR characterize linguistic properties of the transcript that may influence downstream LLM behavior. This is particularly important in clinical speech, where atypical fluency, reduced lexical diversity, and repetition may persist even when word-level recognition appears acceptable. This choice is also consistent with recent work arguing that WER alone is insufficient for evaluating ASR in clinical and atypical speech settings [13]. In addition, these metrics can be computed consistently across all TalkBank datasets without requiring additional task-specific annotations. We also note that alternative metrics such as CER, phonetic similarity, and semantic robustness measures have been proposed in the literature [1, 8, 19], but were not adopted here because our focus is on transcript properties that can be computed consistently across all TalkBank datasets and related directly to downstream LLM performance. "

> "Collectively, these three metrics provide complementary views of summary quality, covering lexical overlap, semantic similarity, and learned human-aligned evaluation. This choice is also consistent with recent evaluation trends in impaired-speech and accessibility benchmarks, which increasingly emphasize semantic quality beyond word-level matching [27]."

4) Summarization Motivation *Why did you choose the prompt of summarization for LLMs? The motivation of this choice seems to be a bit confusing.*

REPLY: We thank the reviewer for raising this important point. We agree that the original manuscript did not sufficiently explain why summarization was chosen as the evaluation task for the LLM component. The reviewer is correct that this rationale should be stated explicitly. In the revised manuscript, we have clarified this motivation and moved the prompt text from the appendix to the main text.

Our choice of summarization is motivated by three considerations.

- **Summarization requires holistic understanding of the transcript.** Our goal is to evaluate how ASR errors affect downstream LLM behavior. Summarization is well suited for this purpose because it requires the model to integrate information across the full transcript, resolve ambiguity, and remain robust to disfluencies, repetitions, and transcription noise. This makes it a stronger test of downstream understanding than tasks based on isolated spans or short responses [14].
- **Summarization is clinically relevant and can be applied uniformly across datasets.** The datasets used in this study consist of clinical conversations and assessment dialogues, for which summarization is a natural downstream task. It also allows a common evaluation protocol across 20 heterogeneous TalkBank datasets without requiring additional task-specific annotations such as question-answer pairs, intent labels, or slot labels [7, 10, 24].
- **Summarization supports robust automatic evaluation at scale.** A study of this size requires a task that can be applied consistently across many heterogeneous datasets without constructing new task-specific labels. Summarization meets this requirement because it can be generated directly from each transcript, and the resulting outputs can be evaluated automatically using established metrics such as ROUGE, BERTScore, and BLEURT. This makes it practical for large-scale evaluation in a way that tasks such as question answering, intent classification, or slot filling would not be.

Taken together, these considerations make summarization a well-justified choice for our study, given the clinical setting, the heterogeneity of the datasets, and the scale of the evaluation. We have revised the manuscript accordingly.

The revised text now reads:

> " We adopt summarization as the evaluation task because it requires holistic semantic understanding of the transcript and is sensitive to transcription errors, disfluencies, and linguistic irregularities in the input. Producing a summary requires the LLM to integrate information across the full conversation, which makes this task more suitable for assessing downstream understanding than tasks based on isolated spans or short responses. Summarization is also clinically relevant for our setting, since patient–clinician

> conversations are commonly distilled into concise notes, and it enables a uniform evaluation protocol across heterogeneous TalkBank datasets without requiring additional task-specific annotations [7, 10, 14, 24]. "

5) Prompt in Main Text *Additionally, the authors should write this prompt into the main text (not only in the Appendix).*
REPLY: We thank the reviewer for this helpful suggestion. We agree that the prompt text should be visible in the main text. In the original submission, the prompt was included in the Methods section, which appears after the reference list may therefore have been easy to overlook. In the revised manuscript, we now reproduce the prompt explicitly in the main text as well, in the opening part of the Results section where the evaluation protocol is introduced.

This revision is intended to make the evaluation setup more transparent and easier to follow. The revised text now reads:

> "Performance is evaluated using three widely adopted semantic similarity metrics, ROUGE, BERTScore, and BLEURT. As ground-truth summaries are not available in the datasets, we construct reference outputs using human-annotated transcripts paired with multiple strong cloud-based LLMs. Specifically, each ASR–LLM processes audio inputs to generate summaries, while reference summaries are generated from human transcripts using a set of high-capability LLMs under the same prompt setting. The prompt used throughout the study is
>
>> *Please summarize the following transcript made by an experiment participant.*
>> *Transcript: <ASR-made/human annotator-made transcript>*
>> Summary:"

2.1 Response to Reviewer 3

1) Contribution and Novelty *The use of existing metrics (ROUGE, BERTScore, BLEURT) in evaluating method and standard statistical tests (Wilcoxon with Bonferroni correction) is not much novel. The novelty appears incremental rather than transformative so the claim should be normalized in introduction. Methodology combines existing techniques; no new algorithms or fundamentally new insights. I suggest authors clarify their contribution in intro. The work may be more suitable for a specialized venue (e.g.,informatics alike) unless the authors can strengthen the novelty and broader impact claims substantially.*
REPLY: We thank the reviewer for this thoughtful and important comment, and for the opportunity to clarify the nature of our contribution. The primary contribution of this work is not algorithmic novelty, but a large-scale, deployment-oriented empirical evaluation of ASR–LLM systems for language-impaired populations, a setting that, to our knowledge, has not previously been studied in a systematic way.

- **On the nature of the contribution.** We agree that the individual evaluation metrics and statistical tests used in this study are established methods. This choice was intentional. We used standard metrics and standard statistical procedures to ensure that the findings are interpretable, reproducible, and comparable with prior work. The novelty of the paper lies in

the problem setting, the evaluation scope, and the deployment-oriented analysis, rather than in proposing a new algorithm or a new metric.

- **On scale, scope, and findings.** Our study evaluates 115 ASR–LLM combinations across 20 TalkBank datasets spanning three clinically distinct populations, Aphasia, language impairments in children, and Dementia. Beyond scale, the study examines ASR and LLM as a chained pipeline under edge-oriented deployment constraints, which differs from prior work that typically evaluates ASR or LLM performance in isolation. This analysis yields several practically relevant findings, including condition-dependent patient–control performance differences, the role of repetition, noise, and transcript length in downstream performance, and the identification of resource-dependent deployment boundaries for long and linguistically complex transcripts.
- **On broader significance and manuscript revision.** We believe the broader significance of the work lies in its implications for inclusive and reliable healthcare AI. Understanding how edge-based ASR–LLM systems behave for communication-impaired populations is important for accessibility, clinical safety, and responsible deployment. Following the reviewer's suggestion, we have revised the Introduction to calibrate the novelty claims more carefully and to position the paper explicitly as a systematic empirical and deployment-oriented study rather than a method paper.

The revised text now reads:

> "In summary, the major contributions of this work are three-fold: 1) To our best knowledge, we present the first large-scale empirical study of ASR–LLM systems for individuals with language impairments, covering multiple clinical populations, datasets, and a broad range of ASR–LLM model combinations under edge-oriented constraints. 2) We systematically characterize how linguistic factors (e.g., repetition, noise, and transcript length) affect ASR–LLM performance, and quantify performance disparities between patients and control groups across clinical conditions. 3) We provide a deployment-oriented analysis of ASR–LLM systems, highlighting the impact of resource constraints such as memory usage, latency, and input length, and identifying practical limitations for real-world edge deployment. We believe that this work provides both a systematic evaluation framework and practical insights for deploying ASR–LLM systems in healthcare settings, particularly for populations with language impairments."

2) GPT-4o as Judge *Currently, a huge debate is going for the use of AI or in your case the GPT-4o as judge, the output on human transcripts as the "reference" or "best case" raises significant methodological concerns. I believe the GPT-4o summaries are not ground truth; they represent one model's interpretation, is there any HITL ? This approach conflates ASR errors with LLM interpretation differences.The authors acknowledge this limitation implicitly but should provide stronger justification or alternative validation.*

REPLY: We thank the reviewer for this important methodological concern. We agree that GPT-4o outputs should not be treated as ground truth in the strict sense, and we have revised the manuscript accordingly. In the revised version, we no longer describe these summaries as "best-case" outputs without qualification, but instead refer to them as reference summaries generated from human transcripts.

To reduce dependence on any single reference model, we expanded the evaluation protocol from a single GPT-4o reference to a multi-reference setting using Claude, GPT-4o, GPT-5.2, and Llama. For each sample, we generated reference summaries from all four models using the human-annotated transcript, computed ROUGE, BERTScore, and BLEURT against each reference separately, and averaged the resulting scores. We then recomputed Tables 1–3 in the original paper under this revised protocol.

This revision led to two important findings. First, some localized changes were observed, most notably in BLEURT for the Aphasia datasets, confirming that single-model references can introduce bias. Second, the overall statistical patterns remained largely unchanged, indicating that the main conclusions of the study are robust to the choice of reference model. To further examine this issue, we also performed a reference-level consistency analysis and observed high agreement across reference models, with Pearson correlations mostly ranging from 0.92 to 0.98 across ROUGE, BERTScore, and BLEURT, as shown in Figure 2.

We agree that human-in-the-loop evaluation would provide valuable additional validation. At the same time, summarization is an inherently open-ended task, and multiple reasonable summaries may exist for the same transcript even under expert review. For a study of this scale, multi-reference evaluation provides a practical and more robust alternative to a single-model reference while remaining tractable. We now discuss this limitation more explicitly in the revised manuscript and cite recent literature supporting the use of LLM-based evaluation for open-ended generation tasks. We also clarify that the reference summaries are always generated from human transcripts, whereas the evaluated ASR–LLM pipeline operates on ASR transcripts, so that ASR-induced variation remains localized to the pipeline under study.

The revised text now reads:

> "To reduce dependence on any single reference model, we construct a multi-LLM reference using four strong cloud-based LLMs, Claude, GPT-4o, GPT-5.2, and Llama. For each sample, each reference model generates a summary from the human transcript using the same prompt setting. We then compute ROUGE, BERTScore, and BLEURT between the ASR–LLM summary and each reference summary separately, and average the resulting scores to obtain the final evaluation score. Higher similarity indicates better ASR–LLM performance, as it suggests that the ASR–LLM output remains closer to reference summaries derived from the human transcript."
>
> "Several limitations should be noted. Although we replaced the original single-model reference with a multi-LLM reference and observed high agreement across reference models, the evaluation still depends on generated summaries instead of human-authored gold summaries."

3) Error Reporting *I am bit confused on the identification of ERROR1 (CUDA out of memory) and ERROR2 (probability tensor errors) in the main paper,these are implementation artifacts rather than fundamental limitations. Modern solutions (gradient checkpointing, flash attention, context windowing) could mitigate these issues.*

REPLY: We thank the reviewer for this helpful observation. We agree that the originally reported error messages were sensitive to implementation details, runtime configuration, and hardware budget, and we appreciate the opportunity to clarify their role in the revised manuscript.

In the revised version, we substantially restructured Section 2.1. The discussion is no longer organized around the labeled error categories themselves, and we no longer use ERROR1 and ERROR2 as the main narrative framing of the section. Instead, the revised section focuses on deployment feasibility across hardware budgets, with emphasis on how long-context inputs affect on-device execution, latency, and energy cost. The specific failure types are now mentioned only briefly in one place, where we note that the observed failures included "CUDA out-of-memory" and "Probability tensor contains either inf, 'nan' or element < 0".

We also agree that techniques such as flash attention, gradient checkpointing, context windowing, and other memory-efficient inference strategies may alleviate some of these issues. We now acknowledge this explicitly in the manuscript. At the same time, these techniques introduce design trade-offs that remain relevant in our setting, including hardware compatibility, additional engineering complexity, and in some cases reduced transcript coverage. For this reason, we view them as important ways to shift the operating boundary, while still considering the observed failure patterns informative for deployment analysis under the studied configuration.

Our additional experiments on physical NVIDIA Jetson AGX Orin 64GB devices help contextualize these observations. Some combinations that produced memory-related failures under the lower-memory setting were able to complete successfully on the AGX platform, showing that the observed failures are hardware-sensitive. However, even on this high-end edge device, long-context inference remained expensive in latency and energy, which suggests that the practical deployment challenge persists even when some memory failures are alleviated.

To reflect this revised interpretation, we removed the original Figures 3 and 4, which focused directly on ERROR1 and ERROR2, and replaced the earlier error-centered presentation with a broader deployment-oriented analysis. We hope this revision makes the manuscript more precise and better aligned with the reviewer's concern.

The revised text now reads:

> "Transcript length showed a strong association with downstream failure behavior. The left panel presents a representative example in which memory demand increases with input length. The middle panel provides a schematic view of how long-context inference can place increasing pressure on the downstream LLM runtime. The right panel shows the population-level trend across token-length bins, where error rates increase monotonically for longer inputs. We note that these observed failure patterns are also influenced by implementation choices, and that memory-efficient techniques such as flash attention, gradient checkpointing, or context windowing may shift the practical boundary at which they arise. Even with this caveat, the broader trend is consistent. Longer transcripts place greater memory and numerical demands on downstream LLM inference and are therefore more likely to trigger failures under constrained deployment settings."

4) Significance Interpretation *I also think that the interpretation of "significance" should be contextualized with clinical meaningfulness.*

REPLY: We thank the reviewer for this important comment. We agree that statistical significance should be interpreted with appropriate caution and should not be treated as an direct indication of clinical meaningfulness.

In this study, statistical significance was tested between patient and control groups across datasets and ASR–LLM configurations. The purpose of these tests was to determine whether the

observed LLM performance differences were systematic. They do not by themselves establish the clinical importance of the observed differences.

We also agree that the clinical meaning of a performance gap depends on the downstream setting in which the system would be used. A statistically significant difference may have different practical implications depending on the task, the users' characteristics, and the level of human oversight in the workflow. For this reason, clinical meaningfulness would require additional validation through clinical workflows.

Following the reviewer's suggestion, we have revised the manuscript to make this distinction clearer. In the Results section, we now refer more explicitly to statistical significance. In the Discussion, we added text noting that the observed differences may have clinical implications for LLM selection, usage, and performance expectations, but that the exact clinical meaningfulness of LLM usage needs to be established and validated at the holistic medical device level in future work. We believe the statistical results remain important because they show that the patient–control differences observed in this study are systematic across multiple datasets (each with different context and participant populations) and model settings. At the same time, we now frame these findings more carefully and avoid interpreting them as direct evidence of clinical significance.

The revised text now reads:

> "A further limitation concerns the interpretation of statistical significance. Several patient–control differences were statistically significant across datasets and model settings, but statistical significance should not be treated as equivalent to clinical meaningfulness. The practical importance of these differences depends on the downstream clinical context, the magnitude of the effect, and whether the resulting transcripts or summaries would influence documentation, interpretation, or care workflows. Establishing clinical meaningfulness will require additional validation through clinician-centered assessment, task-specific clinical criteria, or prospective workflow studies."

5) Ethical Considerations *Please discuss the ethical considerations for deploying potentially biased systems in vulnerable populations.*

REPLY: We thank the reviewer for raising this important point. We agree that the ethical implications of deploying potentially biased ASR–LLM systems in vulnerable populations should be discussed explicitly. In the revised manuscript, we have added a dedicated *Ethical Considerations* subsection to the Discussion.

In this new subsection, we make three points. First, we clarify that the performance differences observed between patient and control groups are not only a technical issue, but also an ethical one, because these systems may be less reliable for the very populations they are intended to support. Second, we discuss the potential clinical consequences of these disparities, including the risk that lower-quality transcripts or summaries may distort clinically meaningful information in downstream workflows. Third, we emphasize several implications for responsible deployment, including population-specific performance audits before clinical use, continued human oversight in any workflow involving AI-generated transcripts or summaries, and the need for more representative benchmark datasets covering diverse language impairment profiles.

Our goal in adding this discussion is to clarify that fairness, reliability, and oversight are essential considerations when these systems are applied to communication-impaired populations. We believe this addition strengthens the manuscript by placing the empirical findings in a broader responsible-AI and healthcare context.

The revised text now reads:

> "The performance gaps observed between patients and controls raise an important ethical concern for healthcare AI deployment. Systems intended to support communication in clinical settings may perform less reliably for the populations that are most likely to benefit from them [21, 25]. In our study, ASR–LLM performance differed between patient and control groups across multiple datasets, which indicates that these systems are not equally robust across populations. This pattern is consistent with prior work showing that speech and language technologies trained predominantly on typical speech often generalize poorly to atypical or impaired speech, in part because such populations remain underrepresented in training data [5].
>
> These disparities have direct clinical implications. Lower-quality ASR transcripts and summaries may distort symptom descriptions, reduce the reliability of clinical documentation, and obscure changes that are meaningful for assessment or treatment. This issue is especially important in language impairment settings, where disfluency, repetition, and reduced lexical diversity are themselves clinically relevant signals. If such signals are handled poorly by the ASR–LLM pipeline, the resulting output may misrepresent the patient's communication and introduce bias into downstream clinical workflows.
>
> These findings suggest that responsible deployment of ASR–LLM systems in healthcare requires population-specific evaluation before clinical use. Performance should be audited separately for patient and control groups, and deployment decisions should account for condition-specific differences in robustness [11]. Human oversight should remain an essential part of any clinical workflow that uses AI-generated transcripts or summaries, particularly for individuals with communication impairments [15]. More broadly, the field would benefit from benchmark datasets that better represent diverse language impairment profiles, severity levels, and demographic backgrounds [5]. The TalkBank resources used here provide an important starting point, but broader dataset development will be necessary for equitable and clinically reliable deployment."

6) ASR Comparison *The comparison of only four ASR models (Figure 9) to select Whisper seems limited. More recent models (e.g., Whisper v3, SeamlessM4T) should be discussed.*

REPLY: We thank the reviewer for this helpful suggestion. We agree that including more recent ASR models would strengthen the justification for selecting Whisper, and we have expanded the comparison accordingly.

In our initial submission, the ASR comparison in Figure 9 evaluated one representative model from each of four ASR families: Canary (NVIDIA), Whisper (OpenAI), Parakeet (NVIDIA/Suno.ai), and Wav2Vec2 (Meta). The intention was to perform a family-level comparison to identify a suitable ASR family for the main experiments, with Whisper-medium serving as the representative model at that stage. We acknowledge that this framing was not sufficiently clear in the original manuscript, which may have made the comparison appear more limited. We have revised the manuscript to clarify this point.

In response to the reviewer's suggestion, we expanded the comparison to include four additional models: Whisper-tiny, Whisper-v3, Wav2Vec2-conformer, and SeamlessM4T. The updated results are shown in Figure 7. The expanded comparison is broadly consistent with our original observations. Across the representative datasets, Whisper-medium and Whisper-v3 show stable

performance and ROUGE scores close to the human-transcript reference baseline. Whisper-tiny demonstrates lower and less stable performance, particularly on the Baycrest and Poler datasets. SeamlessM4T performs competitively in some cases, although it does not consistently outperform the Whisper models across datasets. Wav2Vec2-conformer shows higher variance, particularly for patient groups, which may be less desirable for deployment scenarios requiring robustness across individuals. Notably, Whisper-v3 shows comparable or slightly improved performance relative to Whisper-medium in several datasets. This expanded comparison provides additional support for selecting the Whisper family for the main experiments, while also clarifying the performance characteristics of alternative models.

We have updated the original figure and the corresponding discussion to reflect these additional comparisons and to provide a more complete and transparent justification for the final ASR selection.

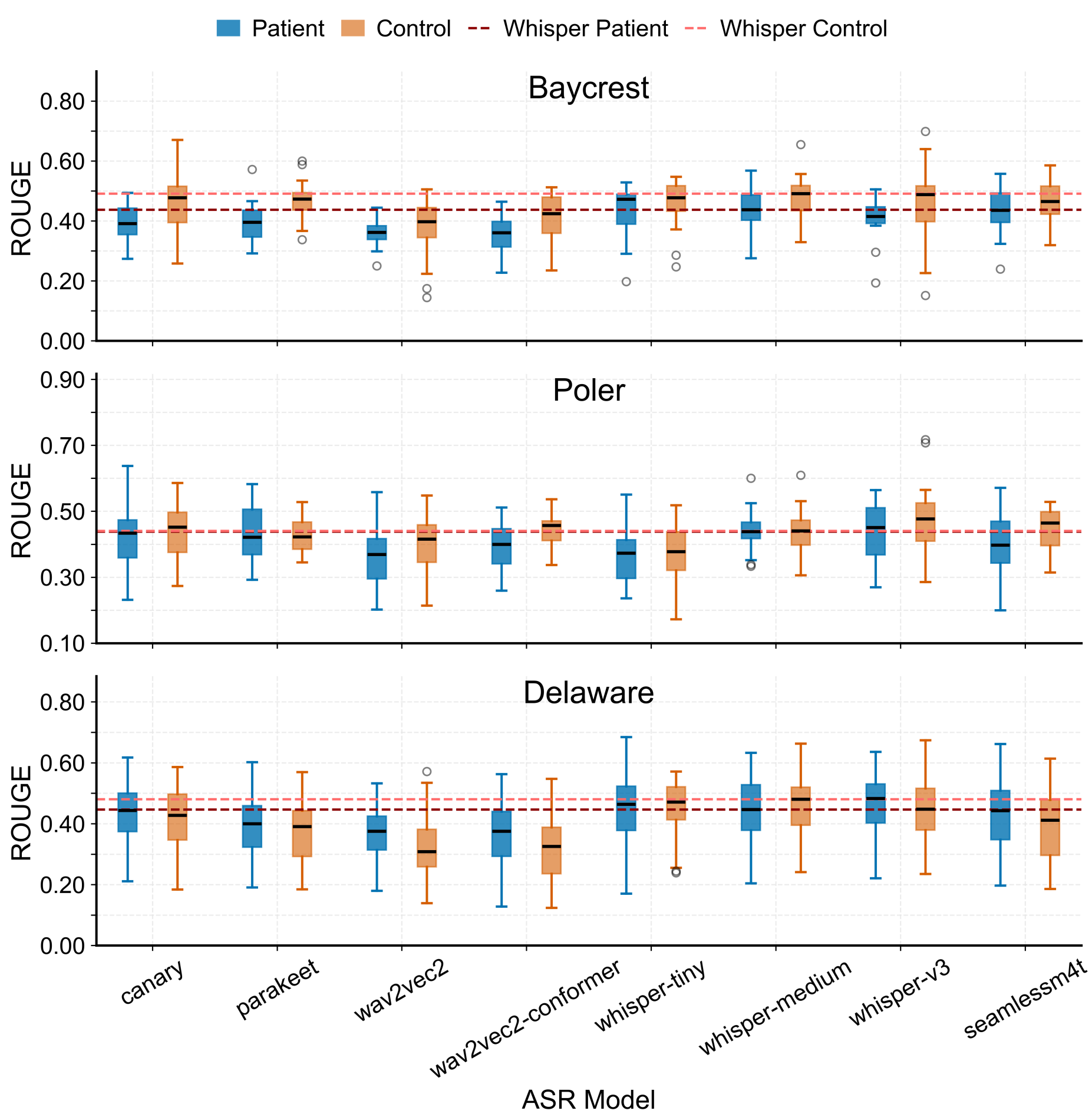

Figure 7: Examine the performance of LLM (Phi-3.5-mini-instruct) on the transcript generated by different types of ASR models.

The revised text now reads:

"For ASR model selection, we compared several widely used ASR models, including

Canary-1B [17] (NVIDIA), Whisper-tiny, Whisper-medium, Whisper-v3 (OpenAI) [20], Parakeet-RNNT-1.1B [18] (NVIDIA), Wav2Vec2-base and Wav2Vec2-conformer [3] (Meta), and SeamlessM4T [4] (Meta). These ASR models were each paired with the same downstream LLM, Phi-3.5-mini-instruct, to generate text from audio on three representative datasets, Baycrest for Aphasia, Poler for LIC, and Delaware for Dementia. As shown in Figure 7, the Whisper family showed the most stable and competitive performance overall across the three datasets. SeamlessM4T performed competitively in some cases, but did not consistently outperform the Whisper models across datasets. Wav2Vec2-conformer also showed higher variability across samples, especially in patient groups. Therefore, Whisper models were selected for use in this study."

7) Sensitivity Analysis *Temperature* $= 0.1$ *and* $top_p = 0.9$ *were used for "stable generation," but no sensitivity analysis was performed on these hyperparameters.*

REPLY:

Dataset		ROUGE		BERTScore		BLEURT	
		Max Std	Max Range	Max Std	Max Range	Max Std	Max Range
Richardson	P	0.0473	0.1103	0.0010	0.0022	0.0447	0.1191
	C	0.0066	0.0148	0.0015	0.0038	0.0088	0.0247
EllisWeismer/66conv	P	0.0107	0.0304	0.0017	0.0041	0.0361	0.0958
	C	0.0102	0.0273	0.0019	0.0055	0.0208	0.0525
ADReSS-IS2020	P	0.0146	0.0460	0.0016	0.0047	0.0303	0.0742
	C	0.0151	0.0391	0.0037	0.0097	0.0268	0.0650

Table 1: Hyperparameter sensitivity analysis.

We thank the reviewer for this helpful suggestion. We agree that the original manuscript did not include a sensitivity analysis for decoding hyperparameters, and we have now added this analysis in the revised manuscript.

Specifically, we conducted a hyperparameter sensitivity analysis across five temperature/top-p combinations ($temperature \in \{0.1, 0.5, 0.9\}$, $top\text{-}p \in \{0.1, 0.5, 0.9\}$), focusing on representative configurations. We evaluated three representative LLMs (gemma-7b-GPTQ, Llama-3.2-3B-Instruct, and Mistral-7B-Instruct-v0.3-GPTQ), and selected one representative dataset from each disease category (Aphasia, Language Impairment in Children, and Dementia). For each setting, we computed the variability of median evaluation scores across ASR–LLM combinations, summarized using the maximum standard deviation (Max Std) and maximum range (Max Range) across all tested configurations.

The results are summarized in Table 1. Overall, we observe that BERTScore remains highly stable across all tested hyperparameter settings, with maximum ranges consistently below or equal to 0.01 across all datasets and groups. ROUGE and BLEURT exhibit slightly larger variability, but the observed changes remain modest and are primarily associated with more extreme decoding configurations (e.g., high temperature or low top-p). Importantly, the variability is generally limited. For example, across all tested datasets, the maximum ROUGE range remains below 0.11, while BLEURT ranges remain below 0.12. In contrast, BERTScore variability remains minimal, with maximum ranges typically below 0.005 and never exceeding 0.01. These results indicate that the semantic similarity of generated summaries is largely robust to decoding hyperparameter variations. We also note that slightly larger variability is occasionally observed in certain patient

datasets (e.g., Richardson), which is consistent with the greater linguistic variability and longer transcript structures observed in these populations. However, even in these cases, the magnitude of variation remains limited and does not qualitatively alter the overall trends.

Taken together, these findings suggest that the original configuration (*temperature* = 0.1, *top-p* = 0.9) represents a conservative and robust choice for stable generation. More importantly, the sensitivity analysis indicates that the main evaluation patterns are not driven by a specific decoding configuration, and that the conclusions of our study remain broadly stable across reasonable hyperparameter variations.

We have added this sensitivity analysis to the revised manuscript and clarified the rationale for our original hyperparameter selection accordingly. The revised text now reads:

> "To verify that the decoding configuration did not unduly affect the main conclusions, we further conducted a sensitivity analysis over multiple $temperature/top_p$ settings, as reported in Table 1. Across representative models and datasets from all three disease categories, BERTScore remained highly stable, with maximum range ≤ 0.01, while ROUGE and BLEURT showed only modest variation under more extreme settings. These results indicate that the selected configuration provides a conservative and robust setting for stable generation."

8) Code *I have seen code, did I miss scripts? it seems to me more results and visuals.*
REPLY: We thank the reviewer for this helpful comment. In response, we have reorganized the codebase and improved the naming of the scripts to make their purposes more transparent. We also added a `README.md` file that explains the function of each script, the overall workflow, and how the main scripts map to the experiments, results, and figures reported in the paper. Our goal in making these changes is to improve clarity and reproducibility, so that readers can more easily identify the scripts used for data processing, model inference, evaluation, and figure generation.

References

[1] Aboeitta, A., Sharshar, A., Nafea, Y., Shehata, S.: Bridging asr and llms for dysarthric speech recognition: Benchmarking self-supervised and generative approaches. arXiv preprint arXiv:2508.08027 (2025)

[2] Agarwal, D., Zhang, H., Yu, Y., Wang, Q.: State-of-the-art dysarthric speech recognition with metaicl for on-the-fly personalization. arXiv preprint arXiv:2509.15516 (2025)

[3] Baevski, A., Zhou, Y., Mohamed, A., Auli, M.: wav2vec 2.0: A framework for self-supervised learning of speech representations. Advances in neural information processing systems **33**, 12449–12460 (2020)

[4] Barrault, L., Chung, Y.A., Meglioli, M.C., Dale, D., Dong, N., Duquenne, P.A., Elsahar, H., Gong, H., Heffernan, K., Hoffman, J., et al.: Seamlessm4t: Massively multilingual & multimodal machine translation. arXiv preprint arXiv:2308.11596 (2023)

[5] Berisha, V., Liss, J.M.: Responsible development of clinical speech ai: bridging the gap between clinical research and technology. NPJ digital medicine **7**(1), 208 (2024)

[6] Brown, P.F., Pietra, V.J.D., Mercer, R.L., Pietra, S.A.D., Lai, J.C.: An estimate of an upper bound for the entropy of english. Computational Linguistics **18**(1), 31–40 (1992)

[7] Chen, Q., Hu, Y., Peng, X., Xie, Q., Jin, Q., Gilson, A., Singer, M.B., Ai, X., Lai, P.T., Wang, Z., et al.: Benchmarking large language models for biomedical natural language processing applications and recommendations. Nature communications **16**(1), 3280 (2025)

[8] Cheng, T.H., Clemmensen, L.H., Das, S.: Beyond word error rate: Auditing the diversity tax in speech recognition through dataset cartography. arXiv preprint arXiv:2603.05267 (2026)

[9] Covington, M.A., McFall, J.D.: Cutting the gordian knot: The moving-average type–token ratio (mattr). Journal of quantitative linguistics **17**(2), 94–100 (2010)

[10] Croxford, E., Gao, Y., Pellegrino, N., Wong, K., Wills, G., First, E., Liao, F., Goswami, C., Patterson, B., Afshar, M.: Current and future state of evaluation of large language models for medical summarization tasks. Npj health systems **2**(1), 6 (2025)

[11] Dankwa-Mullan, I.: Health equity and ethical considerations in using artificial intelligence in public health and medicine. Preventing chronic disease **21**, E64 (2024)

[12] Fraser, K.C., Meltzer, J.A., Graham, N.L., Leonard, C., Hirst, G., Black, S.E., Rochon, E.: Automated classification of primary progressive aphasia subtypes from narrative speech transcripts. Cortex **55**, 43–60 (2014)

[13] Just, S.A., Elvevåg, B., Pandey, S., Nenchev, I., Bröcker, A.L., Montag, C., Morgan, S.E.: Moving beyond word error rate to evaluate automatic speech recognition in clinical samples: Lessons from research into schizophrenia-spectrum disorders. Psychiatry Research p. 116690 (2025)

[14] Liu, Y., Shi, K., He, K., Ye, L., Fabbri, A.R., Liu, P., Radev, D., Cohan, A.: On learning to summarize with large language models as references. In: Proceedings of the 2024 Conference of the North American Chapter of the Association for Computational Linguistics: Human Language Technologies (Volume 1: Long Papers). pp. 8647–8664 (2024)

[15] Mennella, C., Maniscalco, U., De Pietro, G., Esposito, M.: Ethical and regulatory challenges of ai technologies in healthcare: A narrative review. Heliyon **10**(4) (2024)

[16] Müller-Eberstein, M., Yee, D., Yang, K., Mantena, G.V., Lea, C.: Hypernetworks for personalizing asr to atypical speech. Transactions of the Association for Computational Linguistics **12**, 1182–1196 (2024)

[17] NVIDIA: Canary-1b: A multilingual asr/ast model from nvidia nemo. Hugging Face model card (2024), `https://huggingface.co/nvidia/canary-1b`

[18] NVIDIA, Suno.ai: Parakeet-rnnt-1.1b: Fastconformer-transducer asr model from nvidia nemo & suno.ai. Hugging Face model card (2024), `https://huggingface.co/nvidia/parakeet-rnnt-1.1b`

[19] Phukon, B., Zheng, X., Hasegawa-Johnson, M.: Aligning asr evaluation with human and llm judgments: Intelligibility metrics using phonetic, semantic, and nli approaches. arXiv preprint arXiv:2506.16528 (2025)

[20] Radford, A., Kim, J.W., Xu, T., Brockman, G., McLeavey, C., Sutskever, I.: Robust speech recognition via large-scale weak supervision. In: International conference on machine learning. pp. 28492–28518. PMLR (2023)

[21] Rajkomar, A., Hardt, M., Howell, M.D., Corrado, G., Chin, M.H.: Ensuring fairness in machine learning to advance health equity. Annals of internal medicine **169**(12), 866–872 (2018)

[22] Renze, M.: The effect of sampling temperature on problem solving in large language models. In: Findings of the Association for Computational Linguistics: EMNLP 2024. pp. 7346–7356 (2024)

[23] Tan, T., Chen, X., Le, X., Fan, W., Xia, X., Huang, C., Lu, J.: Cba-whisper: Curriculum learning-based adalora fine-tuning on whisper for low-resource dysarthric speech recognition. In: Proc. Interspeech 2025. pp. 3309–3313 (2025)

[24] Van Veen, D., Van Uden, C., Blankemeier, L., Delbrouck, J.B., Aali, A., Bluethgen, C., Pareek, A., Polacin, M., Reis, E.P., Seehofnerová, A., et al.: Adapted large language models can outperform medical experts in clinical text summarization. Nature medicine **30**(4), 1134–1142 (2024)

[25] Xu, G., Duan, Y., Xia, J., Chiu, C.H., Lemmon, M., Jin, W., Shi, Y.: Rethinking fairness in medical imaging: Maximizing group-specific performance with application to skin disease diagnosis. Medical Image Analysis p. 103950 (2026)

[26] Zheng, X., Dong, S., Phukon, B., Hasegawa-Johnson, M., Yoo, C.D.: Towards robust dysarthric speech recognition: Llm-agent post-asr correction beyond wer. arXiv preprint arXiv:2601.21347 (2026)

[27] Zheng, X., Phukon, B., Na, J., Cutrell, E., Han, K., Hasegawa-Johnson, M., Jiang, P.P., Kuila, A., Lea, C., MacDonald, B., et al.: The interspeech 2025 speech accessibility project challenge. arXiv preprint arXiv:2507.22047 (2025)

[28] Zhong, T., Geng, M., Hu, S., Li, G., Liu, X.: Regularized federated learning for privacy-preserving dysarthric and elderly speech recognition. arXiv preprint arXiv:2506.11069 (2025)

Response to Reviewer #1

We thank Reviewer #1 for the careful re-evaluation and for recommending publication. We are grateful for the constructive comments on code availability, which helped us improve the usability and reproducibility of our code supplement.

Remarks on code availability

Comment:
The code is understandable and functional, but lacked proper documentation. Better community access would result from more explanatory content and commentary, including explanations of main functions, model architecture / training pipeline documentation, and a complete README covering installation, dependency versions, dataset preparation, and execution examples.

Response:
We agree with the reviewer and have substantially improved the documentation and readability of the code supplement. Specifically, we have made the following revisions:

1. Expanded README for reproducibility.
   We updated the README to include the essential information requested by the reviewer:
   - installation instructions (`pip install -r requirements.txt`, system dependency `ffmpeg`, recommended Python/GPU setup);
   - dependency versions via a dedicated `requirements.txt`;
   - dataset preparation (paired audio + CHA files and required I/O column schema);
   - step-by-step execution examples for ASR, LLM summarization, metric evaluation, and API baselines;
   - a clear repository map and suggested execution order.
2. Added explanatory comments for main components.
   We added concise module-/function-level documentation for the key scripts, including:
   - `01_asr_cha_segment_transcribe.py` (CHA-aligned Whisper ASR);
   - `02_llm_transcript_summarize.py` (edge/local LLM summarization and OpenAI reference summarization);
   - metric scripts (`Calculate_ROUGE.py`, `Calculate_BLEURT.py`, `Calculate_BERTScore.py`);
   - API baseline scripts (`api_inference-GPT.py`, `api_inference-Claude.py`, `api_inference-Llama.py`).
3. Clarified model usage and pipeline scope.
   Because this work relies on pretrained models for inference and evaluation, we explicitly documented in the README that the supplement focuses on the inference and evaluation pipeline (ASR → summarization → automatic metrics → statistical analysis/figure generation), and does not include model-training code. We also summarized the method workflow and typical decoding settings to help readers understand and replicate the experiments.

The updated code and documentation are publicly available at:
https://github.com/computingx-lab/Clinical-ASR-LLM-Evaluation

We believe these revisions address the reviewer's concerns regarding documentation completeness and community accessibility of the code.
