# Supplementary material for: Assessing the use of automatic speech recognition and large language models for individuals with language impairments
Source: Nat Commun. 2026 Sep 10;17:10716. doi: 10.1038/s41467-026-76677-z (PMC13658231; doi:10.1038/s41467-026-76677-z)
Supplement: Supplementary file 3 [file 41467_2026_76677_MOESM3_ESM.pdf]

nature portfolio

Corresponding author(s): Ruiyang Qin, Yiyu Shi and Zhi Zheng

Last updated by author(s): Apr 29, 2026

Reporting Summary

Nature Portfolio wishes to improve the reproducibility of the work that we publish. This form provides structure for consistency and transparency in reporting. For further information on Nature Portfolio policies, see our Editorial Policies and the Editorial Policy Checklist.

Statistics

For all statistical analyses, confirm that the following items are present in the figure legend, table legend, main text, or Methods section.

n/a	Confirmed	
☐	☒	The exact sample size (*n*) for each experimental group/condition, given as a discrete number and unit of measurement
☐	☒	A statement on whether measurements were taken from distinct samples or whether the same sample was measured repeatedly
☐	☒	The statistical test(s) used AND whether they are one- or two-sided<br>*Only common tests should be described solely by name; describe more complex techniques in the Methods section.*
☒	☐	A description of all covariates tested
☐	☒	A description of any assumptions or corrections, such as tests of normality and adjustment for multiple comparisons
☐	☒	A full description of the statistical parameters including central tendency (e.g. means) or other basic estimates (e.g. regression coefficient) AND variation (e.g. standard deviation) or associated estimates of uncertainty (e.g. confidence intervals)
☐	☒	For null hypothesis testing, the test statistic (e.g. *F*, *t*, *r*) with confidence intervals, effect sizes, degrees of freedom and *P* value noted<br>*Give P values as exact values whenever suitable.*
☒	☐	For Bayesian analysis, information on the choice of priors and Markov chain Monte Carlo settings
☒	☐	For hierarchical and complex designs, identification of the appropriate level for tests and full reporting of outcomes
☐	☒	Estimates of effect sizes (e.g. Cohen's *d*, Pearson's *r*), indicating how they were calculated

Our web collection on statistics for biologists contains articles on many of the points above.

Software and code

Policy information about availability of computer code

Data collection	This study did not involve new primary data collection.
Data analysis	All analyses were implemented in Python using PyTorch and the Hugging Face ecosystem. Code is available at https://github.com/computingx-lab/Clinical-ASR-LLM-Evaluation

For manuscripts utilizing custom algorithms or software that are central to the research but not yet described in published literature, software must be made available to editors and reviewers. We strongly encourage code deposition in a community repository (e.g. GitHub). See the Nature Portfolio guidelines for submitting code & software for further information.

Data

Policy information about availability of data

All manuscripts must include a data availability statement. This statement should provide the following information, where applicable:

- Accession codes, unique identifiers, or web links for publicly available datasets
- A description of any restrictions on data availability
- For clinical datasets or third party data, please ensure that the statement adheres to our policy

This study used existing datasets from TalkBank. All datasets used in this study are available in the TalkBank database. The AphasiaBank datasets are available at https://talkbank.org/aphasia/access/English/Protocol/. The CHILDES Clinical English datasets are available at https://talkbank.org/childes/access/Clinical-Eng/. The DementiaBank datasets are available at https://talkbank.org/dementia/access/ and https://talkbank.org/dementia/ADReSS-2020/.The analyzed data consisted of

audio recordings and professionally human-annotated transcripts from aphasia, language impairment in children, dementia, and corresponding control datasets. Access to the original TalkBank audio recordings and transcripts is governed by TalkBank's data-use terms and the policies of the contributing datasets.

Python 3.10. Model inference and evaluation: PyTorch 2.1–2.4; Transformers 4.40–4.45; Accelerate ≥0.30; Datasets ≥2.19; auto-gptq ≥0.7; bitsandbytes ≥0.43; PEFT ≥0.11. Audio processing: librosa ≥0.10; soundfile ≥0.12; pydub ≥0.25. Evaluation metrics: rouge-score ≥0.1.2; bert-score ≥0.3.13; BLEURT (BLEURT-20 checkpoint). Statistical analysis: SciPy ≥1.11; scikit-learn ≥1.3; NumPy ≥1.24; pandas ≥2.0; openpyxl ≥3.1. Visualization: Matplotlib ≥3.8; seaborn ≥0.13. ASR and LLM models were obtained from Hugging Face.

Research involving human participants, their data, or biological material

Policy information about studies with human participants or human data. See also policy information about sex, gender (identity/presentation), and sexual orientation and race, ethnicity and racism.

Reporting on sex and gender	Sex and gender were not analyzed as variables in this study.
Reporting on race, ethnicity, or other socially relevant groupings	Race, ethnicity, and other socially relevant groupings were not analyzed in this study.
Population characteristics	This study used existing clinical conversational datasets from TalkBank. The analyzed populations included individuals with aphasia, children with language impairments, individuals with dementia, and corresponding control groups.
Recruitment	No new participants were recruited for this study.
Ethics oversight	This study involved secondary analysis of existing TalkBank data and did not involve new participant recruitment.

Note that full information on the approval of the study protocol must also be provided in the manuscript.

Field-specific reporting

Please select the one below that is the best fit for your research. If you are not sure, read the appropriate sections before making your selection.

☐ Life sciences ☒ Behavioural & social sciences ☐ Ecological, evolutionary & environmental sciences

For a reference copy of the document with all sections, see nature.com/documents/nr-reporting-summary-flat.pdf

Behavioural & social sciences study design

All studies must disclose on these points even when the disclosure is negative.

Study description	This was a quantitative secondary computational analysis of existing TalkBank clinical conversational datasets. We evaluated ASR–LLM pipelines for participants with aphasia, language impairments in children, dementia, and corresponding control groups. Audio recordings were transcribed using ASR models and summarized by downstream LLMs, and model outputs were compared with reference summaries generated from human-annotated transcripts.
Research sample	The research sample consisted of existing TalkBank datasets containing audio recordings and professionally human-annotated transcripts. The analyzed populations included participants with aphasia, children with language impairments, individuals with dementia, and corresponding control groups.
Sampling strategy	No new sampling or recruitment was performed
Data collection	No new data were collected for this study.
Timing	No new participant data were collected for this study.
Data exclusions	Datasets were included if they contained both patient and control samples and each sample had corresponding audio recordings and professionally human-annotated transcripts.
Non-participation	Not applicable.
Randomization	Not applicable.

Reporting for specific materials, systems and methods

We require information from authors about some types of materials, experimental systems and methods used in many studies. Here, indicate whether each material, system or method listed is relevant to your study. If you are not sure if a list item applies to your research, read the appropriate section before selecting a response.

Materials & experimental systems

n/a	Involved in the study
☒	☐ Antibodies
☒	☐ Eukaryotic cell lines
☒	☐ Palaeontology and archaeology
☒	☐ Animals and other organisms
☐	☒ Clinical data
☒	☐ Dual use research of concern
☒	☐ Plants

Methods

n/a	Involved in the study
☒	☐ ChIP-seq
☒	☐ Flow cytometry
☒	☐ MRI-based neuroimaging

Clinical data

Policy information about clinical studies

All manuscripts should comply with the ICMJE guidelines for publication of clinical research and a completed CONSORT checklist must be included with all submissions.

Clinical trial registration	Not applicable. This study was not a clinical trial and did not involve any intervention, treatment assignment, or prospective participant recruitment.
Study protocol	Not applicable. This study was a secondary computational analysis of existing TalkBank datasets and did not involve a clinical trial protocol.
Data collection	No new clinical data were collected for this study.
Outcomes	No clinical outcomes were measured or assessed in this study.

Plants

Seed stocks	Not applicable.
Novel plant genotypes	Not applicable.
Authentication	Not applicable.
